# Technological Solutions for Human Movement Analysis in Obese Subjects: A Systematic Review

**DOI:** 10.3390/s23063175

**Published:** 2023-03-16

**Authors:** Riccardo Monfrini, Gianluca Rossetto, Emilia Scalona, Manuela Galli, Veronica Cimolin, Nicola Francesco Lopomo

**Affiliations:** 1 Dipartimento di Ingegneria dell’Informazione, Università degli Studi di Brescia, 25123 Brescia, BS, Italy; 2 Dipartimento di Specialità Medico-Chururgiche, Scienze Radiologiche e Sanità Pubblica, Università degli Studi di Brescia, 25123 Brescia, BS, Italy; 3 Dipartimento di Elettronica, Informazione e Bioingegneria, Politecnico di Milano, 20133 Milano, MI, Italy; 4 Istituto Auxologico Italiano, IRCCS, S. Giuseppe Hospital, Piancavallo, 28824 Oggebbio, VB, Italy

**Keywords:** obesity, human movement analysis, functional assessment, wearable, MIMU, marker-based optoelectronic stereophotogrammetric system, soft-tissue artifacts

## Abstract

Obesity has a critical impact on musculoskeletal systems, and excessive weight directly affects the ability of subjects to realize movements. It is important to monitor the activities of obese subjects, their functional limitations, and the overall risks related to specific motor tasks. From this perspective, this systematic review identified and summarized the main technologies specifically used to acquire and quantify movements in scientific studies involving obese subjects. The search for articles was carried out on electronic databases, i.e., PubMed, Scopus, and Web of Science. We included observational studies performed on adult obese subjects whenever reporting quantitative information concerning their movement. The articles must have been written in English, published after 2010, and concerned subjects who were primarily diagnosed with obesity, thus excluding confounding diseases. Marker-based optoelectronic stereophotogrammetric systems resulted to be the most adopted solution for movement analysis focused on obesity; indeed, wearable technologies based on magneto-inertial measurement units (MIMUs) were recently adopted for analyzing obese subjects. Further, these systems are usually integrated with force platforms, so as to have information about the ground reaction forces. However, few studies specifically reported the reliability and limitations of these approaches due to soft tissue artifacts and crosstalk, which turned out to be the most relevant problems to deal with in this context. In this perspective, in spite of their inherent limitations, medical imaging techniques—such as Magnetic Resonance Imaging (MRI) and biplane radiography—should be used to improve the accuracy of biomechanical evaluations in obese people, and to systematically validate less-invasive approaches.

## 1. Introduction

Obesity represents one of the most prevalent pathologies in developed countries [1], and the global prevalence of obesity has increased by nearly 80% since the 1980s [2].

Obesity has been shown to be a condition that can affect the ability to perform daily-life activities, such as walking [3,4,5,6], lumbopelvic movements [7], standing up [8,9], and tasks involving upper limb movements [10], thus significantly impacting the overall quality of life. In obese individuals, excessive weight can limit and alter their overall capacity for movement, leading to a range of functional limitations [11]. 

Hence, it is important to quantify and monitor how the obese population carries out specific movements. This information can help to characterize their functional limitations, and prevent further issues impacting their musculoskeletal system, due—for instance—to high compressive forces on the anti-gravitational joints and the muscular fatigue [12]. Therefore, there is growing interest in developing tools that are able to monitor the physical activity and movement of the obese population, so as to evaluate the limitations affecting their daily life activities and quantify their clinical conditions.

In this context, it is essential to introduce technological and methodological approaches able to reliably and safely provide quantitative information about the movements of obese subjects. Indeed, the analysis of human motion is widely adopted in clinical and research fields to investigate pathological conditions, including obesity, and pursue an objective and integrated assessment [13]. Specifically, human movement analysis is used to assess the biomechanical features (including both kinematics and kinetics) of obese subjects in order to better understand the most common problems affecting this population.

Currently, movement analysis—particularly motion tracking—is usually performed using marker-based stereophotogrammetric optoelectronic systems integrated with force platforms, and used within controlled experimental environments [13,14]. However, over the past decade, the availability of wearable equipment has made it possible to conduct examinations in more ecological and daily-life conditions [15,16,17,18]. For instance, magneto-inertial measurement units (MIMUs) can provide a quantitative evaluation of movement by integrating information from triaxial accelerometers, gyroscopes, and magnetometers; these solutions are, in general, non-invasive and easy-to-use, allowing for continuous monitoring and self-assessment to evaluate and prevent risk, even at home.

Unfortunately, the use of non-invasive technological solutions that are externally applied to the skin is highly susceptible to soft tissue artifacts (STA) [19], particularly when considering the obese population. This issue can be addressed by simultaneously monitoring the movement of skin markers/sensors and the underlying bone using imaging methods such as biplane radiography [20] and Magnetic Resonance Imaging (MRI) [21].

While numerous studies investigated the application of various technologies to movement analysis in the obese population are present in the literature, a systematic review is currently absent. Therefore, this study aimed to identify and summarize the existing literature, and highlight the different technological and methodological strategies used to evaluate movement analysis approaches within the obesity context, with a particular focus on gait and functional assessment. Based on the outcomes obtained from the reviewing process, this work also aimed to report any advantages or limitations regarding each technology, whenever available and discussed.

## 2. Materials and Methods

### 2.1. Information Sources

This study was conducted and reported following the Preferred Reporting Items for Systematic Review and Meta-Analyses (PRISMA) 2020 [22]. A proper workflow was created following the PRISMA for systematic review protocols (PRISMA-P) [23] which was online, registered on Open Science Framework available at https://doi.org/10.17605/OSF.IO/72UAE (accessed on 5 March 2023). The search process was started in March 2022 by considering three different electronic databases, i.e., PubMed, Scopus, and Web of Science.

Three reviewers (M.R., R.G. and S.E.) were involved in the inclusion/exclusion process, following the eligibility criteria, as hereinafter reported; a fourth reviewer helped in case of discrepancy (L.N.F.).

### 2.2. Eligibility Criteria and Search Strategy

The research question was defined using the Patients-Intervention-Comparison-Outocome-Study (PICOS) approach [24]; in particular, papers were included if they were focused on observational studies reporting quantitative measurements (S); they were focused on obese population (P); they performed the assessment using specific human motion analysis techniques (I). Comparisons (C) and outcomes (O) were not defined.

The full search string was:

*(“obesity” OR “obese”) AND (“biomechanics” OR “movement analysis” OR “motion analysis” OR “wearable” OR “optoelectronic” OR “marker-based” OR “markerless” OR “inertial” OR “IMU”).*

### 2.3. Study Selection

Studies were included if they met the following inclusion criteria:

- published from 2010 to 2022.
- written in the English language.
- conducted on adults (≥18 years old).
- conducted on subjects with Body Mass Index (BMI) > 30.
- reporting at least one measurement parameter/metric related to movement analysis (e.g., joint angles, joint moments, etc.).
- based on observational study design.

Otherwise, the exclusion criteria were:

- Systematic narrative and scoping reviews; letters to editors and commentaries; book or chapters; conference proceedings; case reports or case series.
- Studies including population not stratified among normal-weight, overweight, and obese subjects.
- Studies focused on pre-post intervention evaluation (e.g., arthroplasty, etc.).
- Studies involving patients affected by chronic pathologies and/or patients in pain.
- Studies reporting no information about the used systems/devices/instrumentations (e.g., model or manufacturer specification).
- Studies focused on posture and balance evaluation.

### 2.4. Data Items and Collection

Identified data were exported from electronic databases and imported into a web application for systematic reviews (Rayyan [25]). All the information concerning the studies was manually extracted from the included papers thanks to a full-text analysis, exported, and collected in a custom database, created according to the Cochrane guidelines [26].

As summarized in Table 1, the following data were included:

- Author and year of publication.
- Aim of the work.
- Characteristics of the study population.
- Tasks required of participants.
- Technology used for data acquisition.
- Sensors/marker placement/location.
- Outcomes/measurements.

### 2.5. Quality Assessment: Risk of Bias in Individual Studies

Included papers were assessed using the methodological index for non-randomized studies (MINORS) [27] in order to quantify the individual and overall risk of bias. The MINORS tool comprises a total of twelve items, with items from eight to twelve used only for comparative studies. The items are scored 0 when they are not reported, 1 if they are reported but inadequate, or 2 if reported and adequate. The ideal overall score is 16 for non-comparative studies and 24 for comparative studies. The data extracted from the included studies were summarized qualitatively, providing a narrative synthesis of the findings.

**Table 1:** Study characteristics.

Authors (Year)	Aim of the Work	Study Population	Task	Technology	Sensor Placement	Outcome Measures
Kim et al., 2022 (a) [28]	Investigate changes in whole-body angular momentum in a population with different BMI.	13 obese class 1 and 2 6 M, 7 F BMI 34.1 ± 2.2 kg/m^2^ 11 obese class 3 5 M, 6 F BMI 47.1 ± 7.0 kg/m^2^ 14 normal weights 7 M, 7 F BMI 22.0 ± 2.6 kg/m^2^	Gait on treadmill	Optoelectronic system 10 cameras (Vicon Motion Systems Ltd., Oxford, UK). Split-belt treadmill with 2 force plates (Bertec Corporation, Columbus, OH, USA).	44 passive reflective markers: trunk, pelvis, thighs, shanks, and feet. Markers were placed bilaterally on the posterior heel, three metatarsal heads (1st, 2nd, and 5th), medial and lateral malleoli, medial and lateral femoral epicondyles, greater trochanter, anterior superior iliac spine, posterior superior iliac spinae, and acromion process. A single marker was placed on the xiphoid process, jugular notch, 7th cervical vertebra, and 10th thoracic vertebrae. Rigid clusters of four markers were attached to the shank and thigh bilaterally.	Spatiotemporal gait parameters: walking speed (m/s), step width (m), step length (m), double support time (s). Kinetics: external moment about the body’s COM (Nm), vertical free moment (Nm), whole body angular momentum (Nm).
Kim et al., 2022 (b) [29]	Investigated changes in dynamic balance control in adults with different BMI scores.	14 obese 6 M, 8 F BMI 44.3 ± 7.5 kg/m^2^ 14 normal weights 7 M, 7 F BMI 21.9 ± 2.7 kg/m^2^	Gait overground and on a treadmill	Optoelectronic system 10 cameras (Vicon Motion Systems Ltd., Oxford, UK). Split-belt treadmill with 2 force plates (Bertec Corporation, Columbus, OH, USA). 6.1 m long × 0.9 m wide pressure-sensitive gait carpet (Protokinetics LLC, Peekskill, NY, USA).	44 passive reflective markers: trunk, pelvis, thighs, shanks, and feet. Markers were placed bilaterally on the posterior heel, three metatarsal heads (1st, 2nd, and 5th), medial and lateral malleoli, medial and lateral femoral epicondyles, greater trochanter, anterior superior iliac spine, posterior superior iliac spinae, and acromion process. A single marker was placed on the xiphoid process, jugular notch, 7th cervical vertebra, and 10th thoracic vertebrae. Rigid clusters of four markers were attached to the shank and thigh bilaterally.	Spatiotemporal gait parameters: step width (m), step length (m), double support time (s). Kinetics: whole body angular momentum (Nm), ground reaction force (N), vertical ground reaction moment (Nm), external moment arms (Nm).
Vakula et al., 2022 [30]	Comparison of spatiotemporal parameters and kinetic patterns between young adults with and without obesity.	48 obese 24 M, 24 F BMI 33.0 (32.1–33.9) kg/m^2^ 48 normal weights 24 M, 24 F BMI 21.6 (20.7–22.5) kg/m^2^	Gait overground	Optoelectronic system 9 cameras (Qualisys, Goteborg, Sweden). Two force platforms (AMTI, Watertown, MA, USA).	Calibration: 5 markers were placed on the heel counter, medial and lateral malleoli, and first and fifth meta-tarsals.	Spatiotemporal gait parameters: stance time (s), double support time (s), double support to stance ratio (%), step width (% height), step length (% height), cadence (steps/min), and gait stability ratio (GSR, step/m)). Kinetics: total support moment (Nm), ankle extensor moment (%), knee extensor moment (%), hip extensor moment (%).
Capodaglio et al., 2021 [31]	Quantify the three-dimensional knee and ankle joint kinematics and kinetics in participants with obesity.	32 obese 15 M, 17 F BMI 38.0 ± 4.7 kg/m^2^ 16 normal weights 6 M, 10 F BMI 21.2 ± 2.0 kg/m^2^	Gait overground	Optoelectronic system 6-cameras (460, Vicon Motion Systems Ltd., Oxford, UK) Two force platforms (Kistler Instruments Corp, Winterthur, Switzerland).	Davis, 22 markers [32]	Kinematics: knee and ankle flexion at initial contact (°), maximum ankle dorsiflexion and plantarflexion angles during the stance and swing phase (°), the dynamic range of motion of the knee in the sagittal plane during stance and swing phases (°), the dynamic range of motion for ankle dorsi-plantarflexion in the whole gait cycle (°). Kinetics: maximum value of ankle plantarflexion moment in terminal stance (Nm/kg), the first peak of knee abduction moment and maximum value of knee extension moment (Nm/kg), minimum (W/kg) value in the first phase of stance and maximum (W/kg) ankle power during terminal stance.
Cimolin et al., 2021 [33]	Design and validation of obesity-specific shoes during the walking task with a single IMU.	23 obese 6 M, 17 F BMI > 30 kg/m^2^	Gait overground with and without specific shoes	Single IMU (G-Sensor, BTS Bioengineering, Milan, Italy).	Lower back, approximately at the L4-L5 vertebrae position.	Spatiotemporal gait parameters: walked distance (m), gait speed (m/s), step length (m), and cadence (step/min).
Garcia et al., 2021 [34]	Examine the influence of sex and obesity on sagittal and frontal plane knee mechanics during gait in young adults.	48 obese 24 M, 24 F BMI 33.0 kg/m^2^ 48 normal weights 24 M, 24 F BMI 21.6 kg/m^2^	Gait overground	Optoelectronic system 9 cameras (Qualisys, Goteborg, Sweden). Two force platforms (AMTI, Germantown, MD, USA). Infrared timing gates (Tractronix, Belton, MO, USA).	Passive reflective markers were placed on the lateral aspect of the pelvis to represent the anterior surface of the palpated ASIS landmark. Rigid clusters of 4 non-collinear markers were firmly affixed on the sacrum, and bilaterally on the thigh, shank, and foot segments to minimize soft-tissue artifacts [35].	Kinematics: knee flexion (°) knee varus velocity (°/s). Kinetics: knee adduction moment (Nm), knee flexion moment (Nm).
Ghasemi et al., 2021 [7]	Measurement and comparison between obese and normal-weight subjects of the spine, trunk, pelvis kinematics, lumbopelvic coordination.	9 obese BMI 35.3 ± 2.6 kg/m^2^ 9 normal weights BMI 23.9 ± 1.3 kg/m^2^	Loading handling activities	Opto-electronic system, 10 cameras (Vicon Motion Systems Ltd., Oxford, UK).	Plug in gait, 39 markers [32,36]	Kinematics: trunk, lumbar, and pelvis ROM (°) in all anatomical planes, lumbopelvic ratio (lumbar to pelvis rotations at different trunk angles).
Kim et al., 2021 [37]	Determine the influences of arch height and obesity on gait mechanics in adults with obesity.	26 obese BMI 39.0 kg/m^2^ 21 normal weights BMI 22.7 kg/m^2^	Gait overground and on a treadmill	Opto-electronic system 10 cameras (Vicon Motion Systems Ltd., Oxford, UK). Split-belt treadmill with 2 force plates (Bertec Corporation, Columbus, OH, USA). Pressure-sensitive gait carpet (Protokinetics LLC, Peekskill, NY, USA).	Passive reflective markers were placed bilaterally on the posterior heel, three metatarsal heads (1st, 2nd, and 5th), medial and lateral malleoli, medial and lateral femoral epicondyles, greater trochanter, anterior superior iliac spine, posterior superior iliac spinae, and acromion process. A single marker was placed on the xiphoid process, jugular notch, 7th cervical vertebra, and 10th thoracic vertebrae. Rigid clusters of four markers were attached to the shank and thigh bilaterally.	Spatiotemporal parameters: step width (m), step length (m), double support time (s). Kinematics: knee and ankle joint angle in three anatomical planes (°). Kinetics: knee and ankle peak internal joint moments (Nm).
Law et al., 2021 [38]	Evaluate the difference in lower limbs kinematics and kinetics among 3 groups (normal weight, overweight, and obese) during stair ascent and descent.	11 obese 3 M, 8 F BMI 30.0–34.9 kg/m^2^ 21 overweights 14 M, 7 F BMI 25.9–29.9 kg/m^2^ 20 normal weights 9 M, 11 F BMI 18.5–24.9 kg/m^2^	Stair ascent and descent	Opto-electronic system 10 cameras (Vicon MX-13, Vicon Motion Systems Ltd., Oxford, UK). 4 Force plates: two portable Kistler (Model 9286AA, Kistler Instruments Corp, Winterhur, CH) built into the staircase and two Bertec (Model FP 4060-08, Bertec Corporation, Cloumbus, OH, USA).	Ottawa Motion Analysis Model (UOMAM) [39], 43 markers	Kinematics: Peak and ROM (°) of hip, knee, and ankle angles in the sagittal and frontal plane. Kinetics: peak joint moments (Nm/kg) of hip, knee, and ankle in the sagittal and frontal plane.
Pau et al., 2021 [40]	Assessment of the possible alteration in lower limb joint kinematics in obese individuals during gait.	26 obese 11 M, 15 F BMI Median 39.0 (34.9–51.6) kg/m^2^ 26 normal weights 11 M, 15 F BMI Median 21.4 (17.0–26.5) kg/m^2^	Gait overground	Optoelectronic system 6 cameras (Vicon Motion Systems Ltd., Oxford, UK). Two force platforms (Kistler Instruments Corp, Winterthur, Switzerland).	Davis, 22 markers [32]	Spatiotemporal gait parameters: speed (m/s), stride length (m), cadence (step/min), stance phase (% gait cycle), swing (%gait cycle), double support phase (% gait cycle). Kinematics: range of motion (ROM, °) of hip, knee, and ankle joints in the sagittal plane observed during the gait cycle. Symmetry parameters (Cyclograms): cyclogram area (°^2^), cyclogram orientations (°), trend symmetry.
Maktouf et al., 2020 [41]	Investigate the influence of age and/or obesity on gait parameters, with a focus on ankle muscle activities.	80 obese BMI 37.2 kg/m^2^ 70 normal weights BMI 22.9 kg/m^2^	Gait on treadmill	Gait analysis treadmill with force plates (Zebris; FDM-T, Zebris medical GmbH, Isny, Germany). Surface EMG Powerlab 16/35 system (Powerlab 16/35, ADInstruments, Dunedin, New Zealand).	Two unipodal surface electrodes (Uni-gel Single Electrode-T3425, Thought Technology Ltd., Montreal, Canada) were placed on three ankle joint muscles: the gastrocnemius medialis, the soleus, and the tibialis anterior of the dominant leg.	Spatiotemporal gait parameters: step length (cm), step width (cm), stride length (cm), walking cycle (% gait cycle). Kinetics: CoP length (mm), vertical ground reaction force (N/kg), Root mean square of the gastrocnemius, soleus, and tibialis anterior on the gait cycle.
Sample et al., 2020 [42]	Effects of increased step-width on knee biomechanics during inclined and declined walking.	6 obese 6 M BMI 32.2 ± 2.6 kg/m^2^ 7 normal weights 1 M, 6 F BMI 23.3 ± 2.6 kg/m^2^	Inclined walking	Opto-electronic system 12-cameras (Vicon Motion Systems Ltd., Oxford, UK). Two force platforms (AMTI, BP600600 and OR-6-7; AMTI, Watertown, MA, USA).	Retroreflective anatomical markers were placed on bony landmarks bilaterally on the acromion process, iliac crest, greater trochanter, medial femoral epicondyle, lateral femoral epicondyle, medial malleolus, lateral malleolus, and on the shoe above the first and fifth metatarsal heads and the second toe. For the tracking markers, 4 retroreflective markers, attached to thermoplastic plates, were placed on the posterior trunk, posterior aspect of the pelvis (2 marker clusters on each side), the lateral surface of thighs and shanks, and finally on the the heel of the shoe.	Kinematics: the peak of knee extension/flexion and knee adduction angles (°) and ROM (°). Kinetics: peak loading-response (N), knee extension, and knee abduction moments (Nm).
Badawy et al., 2019 [43]	Evaluation of changes in trunk angles and moments during the dominant side of one-handed carrying of various load.	10 obese M BMI 33.5 kg/m^2^ 10 normal weights M BMI 23.3 kg/m^2^	Gait with different loads in the dominant hand	Opto-electronic system 10 cameras (Vicon Motion Systems Ltd., Oxford, UK). 2 ground force plates (AMTI BP400600, AMTI, Watertown, MA, USA).	Full-body obese specific kinematic marker set, 79 markers [44]	Kinematics: trunk angles (°) in three anatomical planes. Kinetics: the moment at the L4/L5 vertebral segment of the trunk (Nm) in three anatomical planes.
Cimolin et al., 2019 [45]	Validate Time Up and Go test measured by a wearable IMU in obese and normal-weight women.	44 F obese BMI 41.1 ± 7.9 kg/m^2^ 14 F normal weights, BMI 22.8 ± 3.5 kg/m^2^	Time up and go	IMU, (G-Sensor, BTS Bioengineering, Milan, Italy).	Lower back, approximately at the L2 vertebrae position.	Kinematics: trunk flexion/extension angle (°).
Dames et al., 2019 [46]	Comparison of kinematics and kinetics during gait barefoot vs. shod of obese population.	10 obese. 6 M, 4 F BMI 33.7 ± 2.9 kg/m^2^	Gait on treadmill	Optoelectronic system (Vicon Motion Systems Ltd., Oxford, UK). Instrumented treadmill with two force plates (AMTI, Watertown, MA, USA).	Plug in gait [32,36]	Spatiotemporal gait parameters: stride length (m), duration of the stance phase (s), duration of swing time (s), double support time (s). Kinematics: ROM (°) of hip, knee and, ankle joint angles in sagittal plane. Kinetics: joint moment peaks (Nm) of ankle, knee, and hip in sagittal plane.
Pamukoff et al., 2019 [47]	Comparison of femoral cartilage characteristics using ultrasound imaging in individuals with and without obesity.	48 obese 24 M, 24 F BMI (31.9–34.2) kg/m^2^ 48 normal weights, 24 M, 24 F BMI (21.1–22.1) kg/m^2^	Gait overground	Logiq E ultrasound device (GE Healthcare, Fairfield CT, USA) and a 12-5 MHz linear array transducer. Isokinetic dynamometer (HUMAC NORM, Stroughton, MA, USA). Opto-electronic system 9 cameras (Qualisys, Göteborg, Sweden). Two force plates (AMTI, Watertown, MA, USA).	The ultrasound probe was placed anteriorly over the medial and lateral femoral condyles in the transverse plane and superior to the border of the patella. Markers were placed on the lateral side of the pelvis such that the marker represents the anterior surface of the palpated anterior superior iliac spine (ASIS) landmark [48]. Rigid clusters of four markers were affixed to the sacrum, and bilaterally to the thigh, shank, and foot.	Kinetics: peak external knee adduction moment (% BW·ht).
Rosso et al., 2019 [49]	Comparison of gait characteristics of overweight/obese and normal-weight subjects.	10 obese BMI 31.1 ± 3.3 kg/m^2^ 12 normal weights, BMI 22.7 ± 1.2 kg/m^2^	Gait overground	7 IMUs (H-gait, TSDN121, ATR Promotions, Kyoto, Japan).	The sensor on the pelvis was located posteriorly in the middle point between the iliac crests. The six sensors on the lower limbs were positioned on the lateral side of the thighs, on the anterior side of the tibia, and below the medial malleolus, bilaterally [50,51].	Spatiotemporal gait parameters: step length (cm), step width (cm), stride length (cm), cycle time (s), stance time (% gait cycle), and cadence (stride/min). Kinematics: ROM (°) during the gait cycle of the hip, knee, and ankle joint kinematics in the sagittal, frontal, and transversal planes; trajectories of knee and ankle joint center in the transversal plane.
Vakula et al., 2019 [35]	Compare quadriceps function and gait biomechanics in young adults with and without obesity.	48 obese 24 M, 24 F BMI 33.1 ± 4.1 kg/m^2^ 48 normal weights 24 M, 24 F BMI 21.6 ± 1.7 kg/m^2^	Gait overground	Logiq E ultrasound device (GE Healthcare, Fairfield CT) and a 12-5 MHz linear array transducer. Isokinetic dynamometer (HUMAC NORM, Stroughton, MA, USA). Opto-electronic system 9 cameras (Qualisys, Göteborg, Sweden). Two force plates (AMTI, Watertown, MA, USA).	The ultrasound probe was placed anteriorly over the medial and lateral femoral condyles in the transverse plane, and superior to the border of the patella. Markers were placed on the lateral side of the pelvis such that the marker represents the anterior surface of the palpated anterior superior iliac spine (ASIS) landmark [48]. Rigid clusters of four markers were affixed to the sacrum, and bilaterally to the thigh, shank, and foot.	Kinematics: knee flexion and abduction angle (°). Kinetics: vertical ground reaction force (N), vertical loading response (N/s), the external moment of the knee (Nm), knee joint stiffness (Nm per degree).
Clément et al., 2018 [20]	Comparison of soft tissue artifact and its effects on knee kinematics between non-obese and obese subjects performing a squatting activity recorded using an exoskeleton.	8 obese 1 M, 7 F BMI 34.3 ± 2.7 kg/m^2^ 9 normal weights 4 M, 5 F BMI 24.8 ± 2.3 kg/m^2^	Squatting activity wearing an exoskeleton	Exoskeleton (Emovi Inc., Laval, QC, Canada). Biplane radiographic imaging system EOS^®^ system (EOS Imaging Inc., Paris, France).	The exoskeleton was fixed on one of the subjects’ lower limbs and was calibrated to define the anatomical frames of the femur and tibia relative to the technical frames of the exoskeleton. The anatomical frames were built using the functional approach developed by [52].	Kinematics: movement of the femoral harness and tibial plate relative to the femur and tibia; knee flexion angle (°).
Horsak et al., 2018 [48]	Investigate if the test-retest reliability for 3D gait kinematics in a young obese population is affected by using two different hip joint center localization approaches.	10 obese 8 M, 2 F BMI 34.2 ± 3.9 kg/m^2^	Gait overground	Opto-electronic system 8 cameras motion capture system (MX-series, Vicon Motion Systems Ltd., Oxford, UK).	Cleveland Clinic Marker set: twenty-seven retro-reflective spherical markers, some of which were attached in a standardized way as a cluster of three on rigid base plates to the thigh and shank, and others to anatomical landmarks. To account for anterior soft tissue offset of the ASIS markers, the markers were placed on the lateral side of the pelvis, so that the marker center reflects the anterior surface of the palpated ASIS landmark.	Kinematics: hip and knee joint angles (°).
Milner et al., 2018 [53]	Determine how velocity adjustment and different step lengths affect knee joint loading.	10 obese 5 M, 5 F BMI 33.7 ± 3.8 kg/m^2^ 10 normal weights 5 M, 5 F BMI 22.2 ± 1.6 kg/m^2^	Gait overground	Optoelectronic system 8 cameras (Vicon Motion Systems Ltd., Oxford, UK). Two force platforms (AMTI, Watertown, MA, USA).	Anatomical markers: greater trochanters, medial and lateral epicondyles, medial and lateral malleoli, posteroinferior calcaneus, and first and fifth metatarsal heads. Tracking markers: shells located on the posterior pelvis, proximolateral thigh, posterodistal shank, posterosuperior, lateral, and medial aspects of the calcaneus.	Spatiotemporal gait parameters: step length (m) velocity (m/s). Kinetics: peak tibiofemoral joint contact force (N/FFW), tibiofemoral joint impulse (Ns/FFW), peak knee adduction impulse (Nms/FFW·ht).
Yocum et al., 2018 [54]	Investigate self-selected step width and its effects on knee joint biomechanics of obese participants during stair negotiation.	10 obese BMI 32.8 ± 2.7 kg/m^2^ 14 normal weights BMI 22.5 ± 1.9 kg/m^2^	Stair negotiation and walking level	Opto-electronic system 12 cameras (Vicon Motion Systems Ltd., Oxford, UK). Two force platforms (AMTI, BP600600 and OR-6-7, AMTI, Watertown, MA, USA). 3-step staircase (FP-stairs, AMTI, Watertown, MA, USA) bolted to the force platforms.	Retro-reflective anatomical markers were placed bilaterally on the 1st and 5th metatarsal heads, the distal end of 2nd toe, medial and lateral aspects of malleoli and femoral epicondyles, greater trochanters, iliac crests, and acromion processes. A semi-rigid thermoplastic shell with four reflective tracking markers was placed on postero-lateral aspects of the posterior trunk, shanks and thighs, and mid-dorsal aspect of shoes. Four tracking markers placed on two separate shells were placed on the left and right posterior-lateral aspects of the pelvis.	Spatio-temporal parameters: stair ascent, descent, and level walking Step Widths (m) and Speeds (m/s). Kinematics: knee extension and abduction angle (°). Kinetics: vertical ground reaction force (N), knee extension, and knee abduction moments (Nm).
Agostini et al., 2017 [55]	Validation of inertial measurement system for the evaluation of gait parameters in obese and normal-weight population.	10 obese M BMI 31.1 ± 3.3 kg/m^2^ 12 normal weights M BMI 22.8 ± 1.1 kg/m^2^	Gait overground	7 IMUs (H-gait system, TSDN121, ATR Promotions, Kyoto, Japan). Multiple system STEP32: Six footswitches and six electrogoniometers (Medical Technology, Torino, Italy).	Six STEP32 foot-switches were fixed under both barefoot soles (3 under each foot). Footswitches were positioned beneath the back portion of the heel, the first, and fifth metatarsal heads. Six STEP32 electrogoniometric sensors were fixed on the ankle, knee, and hip joints of each lower limb. H-Gait inertial sensors: two below the medial malleolus, two on the shanks in correspondence with the anterior side of the tibia bone, two on the lateral side of the thighs, and one on the pelvis, in the posterior center point between the left and right iliac crest.	Spatiotemporal gait parameters: cadence (strides/min), stance (%gait cycle), swing (%gait cycle), double support (%gait cycle). Kinematics: ROM (°) during the gait cycle of hip, knee, and ankle joint angle in the sagittal plane.
Camomilla et al., 2017 [21]	Assessment of pelvis soft tissue artifact during walking.	1 obese M BMI 36.9 kg/m^2^ 1 overweight M BMI 28.4 kg/m^2^ 3 normal weights 1 M, 2 F BMI 22.3–23.9 kg/m^2^	8 postures: mid-stance postures and star-arc postures	Optoelectronic system 8 cameras (VICON MX, Vicon Motion Systems Ltd., Oxford, UK). MRI (Master Philips Medical System, Best, The Netherlands).	UP-CAST approach matching the point identified on the specific-subject model with marker clusters; Iliac spines, the sacrum, and the right femur lateral and medial epicondyles, 7 markers on the pelvis, 4 markers on the anterior aspect of the thigh [56].	Kinematics: pelvic orientation (°) and joint hip angles (°) in all anatomical planes.
Liu et al., 2017 [57]	Inspect how obesity affects dynamic gait stability among young adults.	23 obese F BMI 35.1 ± 3.9 kg/m^2^ 21 normal weights BMI 21.7 ± 2.4 kg/m^2^	Gait on treadmill	Opto-electronic system 8 cameras (Vicon Motion Systems Ltd., Oxford, UK).	26 retro-reflective markers	Spatio-temporal parameters: step length (m), cadence (step/min), step width (m), gait speed. Kinematics: center of mass (COM), trunk angle (angle between the trunk segment and the vertical axis in the sagittal plane).
Meng et al., 2017 [58]	Assess gait features of normal weight, overweight, and obese adults.	10 obese BMI 35.3 ± 3.1 kg/m^2^ 10 overweight BMI 28.3 ± 1.5 kg/m^2^ 10 normal weights BMI 21.9 ± 1.2 kg/m^2^	Gait overground	7 inertial measurement sensors (Xsens Technologies B.V. Enschede, Netherlands). 4.9 m long GaitRite Portable Walkway System (CIR Systems Inc., Sparta, Netherlands).	Sensors are placed on the sacrum, on the front of bilateral thighs, shanks, and the dorsal surface of the feet.	Spatio-temporal parameters: normalized step length, and normalized stride length. Kinematics: hip and knee joint angle (°).
Singh et al., 2017 [59]	Assess the biomechanical gait changes in obese and normal-weight female adult subjects after a 30-min walking session.	10 obese F BMI 36.1 ± 4.2 kg/m^2^	Gait overground	GaitRite Portable Walkway System (CIR Systems Inc., Sparta, Netherlands). Optotrak motion analysis system (Model 3020; Northern Digital Inc., Waterloo, Ontario, Canada). Kistler force plate (Kistler Instruments Corp, Winterthur, Switzerland).	Triads of infrared-emitting diodes were placed on the pelvis and trunk and bilaterally on the thighs, legs, and feet. Markers were affixed to the lateral aspect of the foot, the shaft of the tibia, and the lateral aspect of the thigh. Femoral epicondyle motion was tracked by two markers mounted on a custom femoral tracking device [60]. Pelvic markers were affixed on the sacrum using a 5-cm extension. A similar extension was placed on the lower cervical vertebrae to track the trunk segment.	Kinetics: hip extension and abduction moment (Nm/kg), knee extension and adduction moment (Nm/kg).
Yang et al., 2017 [61]	Dynamic gait stability control during the slip differs between obese and normal- weight young adults.	23 obese 15 M, 8 F BMI 35.1 ± 3.9 kg/m^2^ 20 normal weights 6 M, 14 F BMI 21.6 ± 2.4 kg/m^2^	Perturbed gait on a treadmill	ActiveStep treadmill (Simbex, Lebanon, NH, USA) was donned with a safety harness instrumented with a load cell. Opto-electronic system (Vicon Motion Systems Ltd., Oxford, UK).	26 retro-reflective markers	Kinematics: trunk angle (°). Kinetics: knee joint isometric strength capability in flexors and extensors (Nm/kg); COM stability, position, and velocity.
Pamukoff et al., 2016 [62]	Compare gait biomechanics between normal-weight and obese young adults.	15 obese BMI (30.2–36.7) kg/m^2^ 15 normal weights BMI (21.0–22.1) kg/m^2^	Gait overground	Electromagnetic tracking sensors (Motion Star, Ascension Corp., Burlington, VT, USA) acquired by the Motion Monitor motion capture system (Innovative Sports Training, Chicago, IL, USA). Non-conductive force plate (Model 4060-NC, Bertec Corp., Columbus, OH, USA).	Sensors were positioned on the pelvis, thigh, shank, and foot of the dominant limb [63].	Kinematics: the peak of knee flexion angle (°). Kinetics: vertical ground reaction force (N/bw), knee internal and external moment (bw·ht)
Fu et al., 2015 [64]	Quantify the effect of obesity on soft tissue work during level walking at a constant velocity.	11 obese BMI 34.9 ± 4.1 kg/m^2^ 9 normal weights BMI 22.0 ± 1.0	Gait on treadmill	Opto-electronic system 7 cameras (Nexus, Vicon Motion Systems Ltd., Oxford, UK). Split-belt force-measuring treadmill (Bertec Corp, Columbus, OH, USA).	Passive reflective markers were placed over the seventh cervical vertebrae, acromion processes, right scapular inferior angle, sternoclavicular notch, xiphoid process, 10th thoracic vertebrae, posterior-superior iliac spines, medial/lateral femoral epicondyles, medial/lateral malleoli, calcanei, first metatarsal heads, second metatarsal heads, and proximal and distal heads of the fifth metatarsals. To account for adipose tis- sue around the pelvis, virtual markers were placed on the anterior superior iliac spines and iliac crests using a digitizing wand (C-Motion, Germantown, MD). Marker clusters were placed on the thighs, shanks, and sacrum to aid in 3D tracking [44].	Kinematics: sagittal component of hip, knee, and ankle angles (°). Kinetics: the sagittal moment of hip, knee, and ankle (Nm/kg), power of hip, knee, and ankle (W/kg).
Singh et al. , 2015 [65]	Analyze the biomechanics of obese and normal-weight females during squat and lunge exercises.	10 obese F BMI 39.2 ± 3.7 kg/m^2^ 10 normal weights F, BMI 21.6 ± 2.3 kg/m^2^	Squat and lunge exercise	Triads of infrared emitting diodes (IREDs) Optotrak motion analysis system (Model 3020, Northern Digital Inc., Waterloo, ON, Canada). Kistler force plate (Kistler Instruments Corp., Winterthur, Switzerland).	Markers were affixed to the lateral aspect of the foot, to the shaft of the tibia, and the lateral aspect of the thigh. Femoral epicondyle motion was tracked by two markers mounted on a custom femoral tracking device [66]. Pelvic and trunk marker triads were attached to 5 cm extensions with base plates affixed over the sacrum and lower cervi- cal vertebrae.	Kinematics: range of motion at the hip, knee, and ankle, and trunk segment flexion angles (°). Kinetics: support moment (summation of the lower limb ankle, knee, and hip extensor moments, Nm/kg).
Glave et al., 2014 [67]	Examine how the classification of participants by body fat percentage resulted in a different change to select kinematic variables during gait in the female population.	12 obese F BMI 31.4 ± 7.0 kg/m^2^ 10 normal weights F, BMI 21.7 ± 2.1 kg/m^2^	Gait overground	Peak motus motion analysis software (Vicon Motion Systems Ltd., Oxford, UK) 2 cameras (frontal and lateral perspective)	6 reflective markers for sagittal view: anterior superior iliac spine, the external border of the greater trochanter, the lateral epicondyle of the femur, the lateral malleolus, the base of the fifth toe, and the back of the heel. 3 markers for frontal view: 1 on the sacrum and on both heels.	Spatiotemporal gait parameters: walking speed (m/s), stride length (m), step width (m). Kinematics: knee angular displacement (°), ankle angular displacement (°), peak knee flexion velocity (°/s), peak knee extension velocity (°/s).
Haight et al., 2014 [68]	Examine how different walking conditions reduced tibiofemoral loading.	9 obese 1 M, 8 F BMI 35.0 ± 3.8 kg/m^2^ 10 normal weights 5 M, 5 F BMI 22.1 ± 1.0 kg/m^2^	Walking at different speeds and inclinations on a treadmill	Optoelectronic system 10 cameras (Nexus, Vicon Motion Systems Ltd., Oxford, UK). Dual belt, inclinable, force-measuring treadmill (Fully instrumented treadmill; Bertec Corp., Columbus, OH, USA). Surface EMG (Noraxon, Scottsdale, A, USA).	Obesity-specific marker set [44] EMG electrodes: soleus, lateral gastrocnemius, vastus lateralis, vastus medialis, biceps femoris long head, semimembranosus.	Kinematics: the peak of hip, knee, and ankle flexion/extension angles (°). Kinetics: the peak of hip extension, knee extension, internal abduction, ankle dorsiflexion moments; peak compressive tibiofemoral forces, and rates of tibiofemoral loading.
Lerner et al., 2014 (a) [69]	Evaluate the effect of different speeds on lower limb muscles.	9 obese 1 M, 8 F BMI 30-40 kg/m^2^ 10 normal weights 5 M, 5 F BMI < 25 kg/m^2^	Walking at different speeds on a treadmill	Optoelectronic system 10 cameras (Nexus, Vicon Motion Systems Ltd., Oxford, UK). Dual belt, inclinable, force-measuring treadmill (Fully instrumented treadmill; Bertec Corp., Columbus, OH, USA). Surface EMG (Noraxon, Scottsdale, A, USA).	Obesity-specific marker set [44] EMG electrodes: soleus, lateral gastrocnemius, vastus lateralis, vastus medialis, biceps femoris long head, semimembranosus.	Kinematics: the peak of the pelvis, hip, knee, and ankle flexion/extension angles (°). Kinetics: individual muscle forces (from EMG data) and individual muscle contribution (ground reaction force).
Lerner et al., 2014 (b) [44]	Developed an obesity-specific marker set that accounted for subcutaneous adiposity.	9 obese 1 M, 8 F BMI 35.0 ± 3.8 kg/m^2^ 10 normal weights 5 M, 5 F BMI 22.1 ± 1.0 kg/m^2^	Walking on treadmill	Optoelectronic system 10 cameras (Nexus, Vicon Motion Systems Ltd., Oxford, UK). Dual belt, inclinable, force-measuring treadmill (Fully instrumented treadmill; Bertec Corp., Columbus, OH, USA). Surface EMG (Noraxon, Scottsdale, A, USA).	Obesity-specific marker set which utilized digitized markers and marker clusters and a modified Helen Hayes marker set. EMG electrodes: soleus, lateral gastrocnemius, vastus lateralis, vastus medialis, biceps femoris long head, semimembranosus.	Kinematics: sagittal plane joint angles of the pelvis, hip, and knee. Kinetics: axial joint contact force of hip and knee; muscle force of vasti, hamstring, rectus femoris, and iliopsoas.
Mignardot et al., 2013 [70]	Identifying the role and contribution of some morphological characteristics and the physical activity lifestyle in the observed postural-kinetic deficits.	12 obese 5 M, 7 F BMI 36.6 ± 3.3 kg/m^2^ 8 normal weights 4 M, 4 F BMI 21.4 ± 2.0 kg/m^2^	Gait overground	Optoelectronic system (optotrack 3020 H, NDI, Ontario, Canada). Force platform (OR6-7, AMTI, Watertown, MA, USA).	Ten active markers were placed on anatomical landmarks: eyes, ear (auditory meatus), shoulder (acromion), elbow (ulnar epicondyle), wrist (radial tuberosity), finger (head of the 5th metacarpal bone), hip (greater trochanter), knee (lateral femoral condyle), ankle (lateral malleolus), and foot (fifth metatarsal head).	Kinematics: wrist mean speed (m/s), range of motion of elevation angle of the shank, thigh, pelvis, head, humerus, forearm, hand (°); intersegmental angle shift of ankle, knee, hip, neck, shoulder, elbow, and wrist (°). Kinetics: center of mass (COM) and center of pressure (COP) in three anatomical directions.
Ranavolo et al., 2013 [71]	Evaluate how obesity affects coordination during locomotion using the CRP (continuous relative phase) method.	25 obese 8 M, 17 F BMI (33.8–44) kg/m^2^ 25 normal weights 8 M, 17 F BMI (19.0–27.8) kg/m^2^	Gait overground	Opto-electronic system 8 cameras (SMART–E System, BTS Bioengineering, Milan, Italy).	Davis marker placement protocol [32].	Spatio-temporal gait parameters: stride duration (s), mean speed (m/s), cadence (step/min), duration of the stance (%), duration of the swing (%), and double support phase (%). Kinematics: range of motion of pelvic tilt, obliquity, and rotation; hip flexion/extension, abd/add, rot; knee flexion/extension, ankle dorsi/plantarflexion, foot progression, inter-joint coordination on the sagittal plane.
Roemer et al., 2013 [72]	Determine the effects of BMI on the biomechanics of ergometer rowing in the lower extremities.	10 obese BMI 35.5 ± 4.7 kg/m^2^ 10 overweights BMI 26.7 ± 1.3kg/m^2^ 10 normal weights BMI 21.8 ± 1.6 kg/m^2^	Rowing	Opto-electronic system 6 cameras (Vicon Motion Systems Ltd., Oxford, UK). Concept II Model D ergometer equipped with two 3D AMTI force transducers (AMTI, Watertown, MA, USA).	48 passive reflective markers	Kinematics: flexion/extension and ab-/adduction angles and torques in hip, knee, and ankle joint; internal/external rotation angles (°). Kinetics: torques of hip and knee joint.
Russel et al., 2013 [73]	Determine if laterally wedged insoles could reduce the peak knee joint contact force and the peak medial location of the joint contact force. during walking in obese women.	14 obese F BMI 37.2 ± 6.1 kg/m^2^ 14 normal weights F BMI 22.4 ± 1.2 kg/m^2^	Gait overground	Opto-electronic system 8 cameras (240 Hz; Oqus 300, Qualisys, Gothen- burg, Sweden). One force platform (AMTI, Watertown, MA, USA).	Spherical retro-reflective markers were placed on the pelvis including the iliac crests, greater trochanters, anterior superior iliac spines, and the space between the fifth lumbar and first sacral vertebrae. Posterior superior iliac spine markers were used to help track the motion of the pelvis in the obese group as markers on the anterior superior iliac spines can experience excessive movement in this population. Other markers were secured to the medial and lateral femoral epicondyles and malleoli. Locations on the foot were palpated through the shoe and included the first and fifth metatarsal heads and the distal toe. Rigid arrays of markers were secured to the lateral thigh, lateral leg, and posterior heel.	Kinematics: hip, knee, and ankle 3D joint angles (°). Kinetics: ground reaction forces, 3D hip moment, knee flexion/extension moment, and ankle subtalar and talocrural moment (Nm); Center of pressure lateral and medial (cm).
Silvernail et al., 2013 [74]	Influence of BMI and velocity on knee biomechanics in walking.	10 obese BMI 34.4 ± 3.9 kg/m^2^ 10 overweights BMI 26.9 ± 1.3 kg/m^2^ 10 normal weights BMI 22.4 ± 2.1 kg/m^2^	Gait overground	Opto-electronic system 7 cameras (Vicon Motion Systems Ltd., Oxford, UK). Two force platforms (AMTI, Watertown, MA, USA).	Anatomical markers were placed bilaterally on the iliac crest, greater trochanter, medial and lateral epicondyles, medial and lateral malleoli, and the first and fifth metatarsal heads. Four non-collinear tracking markers were attached to molded thermoplastic shells [75] on the pelvis, thighs, and shanks [30], and three separate non-collinear markers on the heels [76].	Spatio-temporal parameters: walking velocity (m/s). Kinematics: knee flexion excursion (°), peak knee flexion angle (°), knee adduction angle (°). Kinetics: external knee adduction moment (Nm/ffw·ht), external knee flexion moment (Nm/ffw·ht).
Ehlen et al., 2011 [77]	Quantify the energetics and biomechanics of uphill versus level walking in moderately obese adults.	12 obese 5 M, 7 F BMI 33.4 ± 2.4 kg/m^2^	Inclined and level walking on a treadmill	Three-dimensional motion capture system (Motus 9.2; Vicon Motion Systems Ltd., Oxford, UK). Dual-belt, inclinable, force-measuring treadmill (Fully Instrumented Treadmill; Bertec, Columbus, OH, USA).	Retro-reflective spheres following the modified Helen Hayes marker set to identify anatomical landmarks and delineate lower extremity segments [36]. Markers were placed on the sacrum and anterior superior iliac spine, mid-thigh (femoral wand), femoral epicondyle, mid-shank (tibial wand), lateral malleolus, second metatarsal head, and calcaneus of each leg.	Kinematics: hip, knee, ankle sagittal joint angle (°). Kinetics: ground reaction force (N), hip and knee extension, knee abduction, and ankle plantarflexion moment (Nm).
Russel et al., 2010 [78]	Metabolic cost and biomechanical risk factors for the knee in obese women.	10 obese F BMI 33.1 ± 4.2 kg/m^2^ 10 normal weights F BMI 22.7 ± 0.9 kg/m^2^	Gait overground and on the treadmill	Opto-electronic system 8 cameras Qualisys Mo- tion Capture System (Qualisys, Gothenburg, Sweden). Three-dimensional force platform (AMTI, Watertown, MA, USA).	Marker locations included bilateral iliac crests, greater trochanters and anterior superior iliac spines. A sacral marker was placed on L5/S1. Other markers on the right leg only included medial and lateral femoral epicondyles, medial and lateral malleoli, first and fifth metatarsal heads, and the distal end of the first metatarsal. Rigid arrays of markers secured to the right lateral thigh, lower leg, and posterior heel tracked the motion of the segments.	Kinematics: peak impact shock during foot-ground contact (vertical deceleration m/s^2^). Kinetics: peak adduction moment of the knee (Nm) and knee adduction moment angular impulse (Nm).
Vismara et al., 2010 [79]	Proposal of a protocol to evaluate the functional mobility of the spine segment.	13 obese F BMI 39.2 ± 3.6 kg/m^2^ 13 obese with non-specific low back pain F BMI 41.9 ± 5.3 kg/m^2^ 11 normal weights F BMI 20.1 ± 1.2 kg/m^2^	Forward flexion and lateral bending of the trunk	Optoelectronic system 6 cameras (Vicon 460, Vicon Motion Systems Ltd., Oxford, UK).	Five markers were placed along the spine: two on the thoracic (T1 and T6), two on the lumbar vertebrae (L1 and L3), and one on the sacrum (S1). Four markers on the pelvis: left and right anterior and left and right posterior iliac spines (LASIS, RASIS, LPSIS, RPSIS). Two markers on the acromion of the left and right shoulders.	Kinematics: sagittal plane: forward trunk inclination, anterior pelvic tilt, angle related to lordosis, lumbar movement, angle related to kyphosis, thoracic movement. Frontal plane: lateral bending, lateral trunk inclination, pelvis obliquity, lumbar curve, lumbar movement, thoracic curve, thoracic movement, and shoulders. Symmetry index of lateral trunk inclination.

## 3. Results

### 3.1. Study Selection

**Figure 1:**
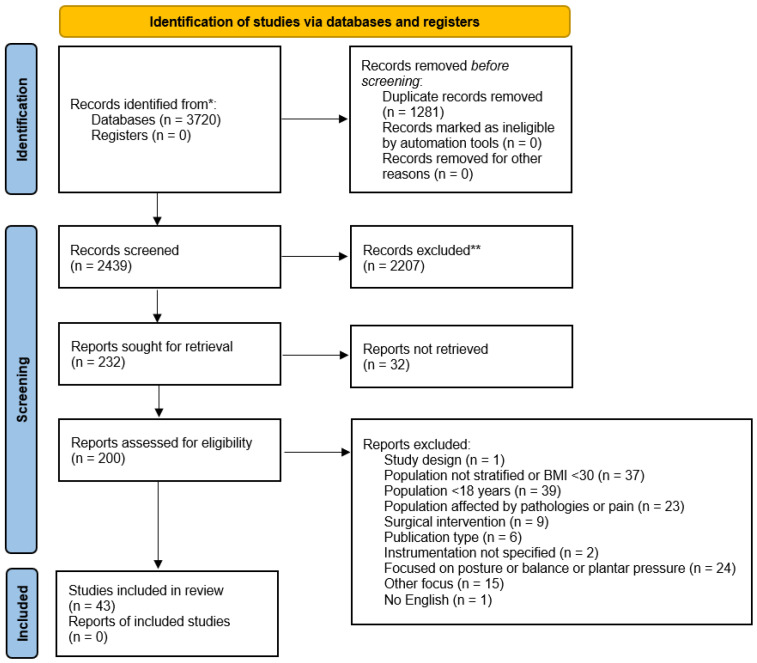
PRISMA diagram. * PubMed = 913; Scopus = 1659; Web of Science = 1148; ** manually excluded.

The initial search identified a total of 3720 papers; of these, 1281 were duplicates and were removed accordingly. The obtained 2439 studies were preliminary screened for eligibility criteria, and 2207 were eliminated. A total of 232 were then screened in full, and from the residual papers: thirty-two had full text not available; one had a different study design; thirty-seven had a population not stratified according to the inclusion criteria or with BMI < 30 kg/m^2^; thirty-nine had a population with less than 18 years; twenty-three included a population affected by other pathologies or pain; nine were focused on the surgical intervention; two papers had no precise indication about the instrumentation; twenty-four papers were focused on posture or balance, or the analysis of plantar pressure; fifteen papers had other focuses; one paper was not written in English; six papers were different publication types from the inclusion criteria. Finally, a total of 43 papers were included in this review. The complete PRISMA flowchart is reported in Figure 1.

In the Supplementary Materials, a detailed description of the references and the reasons for exclusions are reported.

### 3.2. Risk of Bias

Focusing on the evaluation of risk of bias, Table 2 shows the scores specifically associated with the included articles and estimated through the MINORS tool [37].

Items 1 (clearly stated aim), 3 (prospective collection of data), and 4 (endpoints appropriate to the aim of the study) were graded as two in all forty-three studies. Item 2 (inclusion of consecutive patients) represented a critical item since all studies were graded as one. Item 5 (unbiased assessment of the study endpoint) was also considered critical, and was graded as zero in all studies because no one mentioned strategies or methods to avoid bias concerning this issue. Items 6 and 7 were graded as zero in all forty-three studies because no studies had a follow-up evaluation in their study design.

A further critical item was represented by Item 8, where twenty-one papers were graded as zero, and one paper was graded as one. Item 9 (adequate control group) was graded as two when the control group was available, and the BMI stratification was used as a reference. Item 10 (contemporary groups) was graded as two in thirty-three studies, where subjects were collected for this study; this point was graded as one in five studies, where data were already available and derived from retrospective studies.

Furthermore, Item 11 (baseline equivalence of groups) was graded as two in twenty-nine studies, where samples were balanced between the study group and the control group; this item was graded as one in nine studies because the data were reported but considered not adequate. Finally, Item 12 (adequate statistical analysis), where twenty-three of thirty-eight papers were graded as one, and fifteen papers were graded as two.

### 3.3. Study Characteristics

Study characteristics are summarized and displayed in Table 1.

Considering the population involved, five of forty-three papers included only one group of obese subjects without a control group [33,46,48,59,77]; six studies included three groups, represented by obese, over-weight, and normal-weight subjects [21,38,58,72,74,79]. The remaining thirty-two papers involved two groups, i.e., obese and normal-weight subjects. 

More than half of the papers focused on gait tasks: (i) gait overground [30,31,33,34,35,40,47,48,49,53,55,58,59,62,67,70,71,73,74]; (ii) gait on the treadmill [28,41,44,46,57,64]; and (iii) the comparison between the overground and treadmill [29,37,78]. Moreover, perturbed gait [61], inclined walking [42,68,77], and gaits with different loads in the dominant hand [43] were investigated. Only in a few papers were subjects asked to perform more functional movements: stair ascending and descending [38,54], spinal monitoring during load handling activities [7], squatting activities [20,65], different pelvis postures such as mid stance postures, star arc postures, and other postures [21], time up and go [45], rowing [72], and forward flexion and lateral bending of the trunk [79]. 

The most adopted technology for movement analysis even in obese subjects resulted to be the marker-based optoelectronic stereophotogrammetric systems [7,21,48,57,71,79], usually integrated with two or more force platforms [28,29,30,31,34,35,37,38,40,42,43,44,46,47,53,54,57,59,64,65,68,69,70,72,73,74,77,78].

Passive refracting markers were usually located according to standard protocols, such as “Davis”, “Plug-in Gait”, or “Hellen Hayes” [31,40,46,77], while different custom marker-sets were also proposed according to the different objectives. The relevant anatomical markers position for the gait assessment were iliac spines, greater trochanters, medial and lateral epicondyles, medial and lateral malleoli, lateral wands over the mid-femur and mid-tibia, medial and lateral knees, medial and lateral ankles, second metatarsal heads of the toes, and heels. Lerner et al., [44] proposed a market-set specifically designed for assessing movement in obese population, which was also adopted in [43,68,69].

In this context, Camomilla et al., [21] quantified the soft tissue displacement of pelvic landmarks during hip movements integrating the data gathered from the optoelectronic system and MRI, while Clement et al., [20] evaluated the effects of STA on the estimated 3D knee kinematics using biplane radiography.

Concerning wearable technologies, in five papers, MIMUs were specifically adopted, focusing on a single sensor [33,45] or using a 7-sensors protocol for assessing only the lower limb kinematics [49,55,58]. Pamukoff et al., [62] used electromagnetic tracking sensors, while Clement et al., [20] used an exoskeleton to estimate the kinematics of the lower limbs. 

Surface Electromyography (sEMG) was further adopted in [41,44,68,69] to quantify the muscle activity and estimate the residual lower-extremity muscle forces in the obese population. 

In 17 papers spatiotemporal gait parameters were evaluated [28,29,30,33,37,40,41,46,49,53,54,55,57,58,67,71,74], and Pau et al., [40] also studied the gait symmetry. Thirty-five papers evaluated kinematic parameters such as the hip, knee, and ankle joint angles, trunk and pelvic segments, and spinal range of motion [7,20,21,31,34,35,37,38,40,42,43,44,45,46,48,49,54,55,57,58,61,62,64,65,67,68,69,70,71,72,73,74,77,78]. Finally, 29 papers evaluated kinetic parameters [28,30,31,34,35,37,38,40,41,42,43,44,46,47,53,54,59,61,62,64,65,68,69,70,72,73,74,77,78,80].

Concerning sEMG data, the root means the square of muscle activation signal during the gait cycle was evaluated in [41], whereas the peak of compression tibiofemoral forces and rate of tibiofemoral loading in [68], and the individual muscle forces in [44,69].

## 4. Discussion

In this systematic review, we identified and summarized the evidence provided by 43 original studies regarding the different technological and methodological strategies used to assess the human movement in obese subjects. To our knowledge, no systematic reviews on this topic were published so far.

Upon analyzing the literature and examining available technologies for human movement analysis, it appears evident that the majority of the adopted solutions are marker-based optoelectronic stereophotogrammetric systems. However, it is worth noting that most of the studies that use motion capture systems for gait analysis—even those directly assessing the impact of obesity—use standard kinematic marker-sets or methodologies developed for non-obese individuals, thus not accounting for adiposity; these approaches were, namely, “Plug-in Gait”, “Davis”, and versions of the “Helen Hayes” marker-set [32,36]. Although the literature confirms that it is extremely difficult to identify anatomical landmarks in obese individuals by using a palpatory approach [81], markers are usually placed on the skin trying to guess the placement in correspondence with the underlying anatomical references of interest [82], but without quantifying the error or estimating the possible impact on the final outcomes. In fact, the kinematic data derived from a (skin) marker-based motion capture system are extremely vulnerable to soft tissue artifacts (STAs) [19], which are caused by the relative movement of the tissues over the underlying bone reference [21]. It is evident that obese subjects are more sensitive to STAs, due to the presence of adipose tissue, which can thence increase the error in estimating the overall kinematics. In order to mitigate this issue, several researchers used clusters of markers fixed to rigid plates, aiming at reducing the effect of skin movement by limiting relative marker motion [21,28,29,34,35,37,42,44,47,48,64]. Furthermore, a study presented an obesity-specific motion capture methodology that used a spring-loaded digitizing pointer to manually mark the ASIS landmarks and an additional marker on the iliac crest, which are used to define the pelvis segment [44]. However, the massive amount of STAs characterizing obese populations warrants further investigation of the overall accuracy of marker-based approaches, depending on the specific tasks and setup.

To further counteract this problem, several studies proposed medical imaging approaches oriented to quantifying STAs and their effects and impacts on the kinematics of obese individuals. Among these studies, one was specifically focused on the use of MRI for studying STAs at the pelvis [21], which indeed represents the most critical segment due to the presence of the waist adipose tissue; however, the analysis was conducted on a small cohort of subjects who were classified as obese. Another research specifically analyzed knee kinematics during squatting while the subjects wore an exoskeleton, and STAs were estimated via biplane radiography [20]. Analyzing these papers, we can affirm that medical imaging approaches indeed returned the most reliable information directly focused on bone motions; for this reason, they can be considered as the basis to validate other methodologies. On the other hand, they are in fact invasive (e.g., ionizing radiation), difficult to use due to their inherent complexities, expansive, and bulky to be used in daily inpatient/outpatient setups or during daily clinical practice. Furthermore, to provide reliable information, these approaches require biomechanical subject-specific models, which require additional resources to be defined and reliably implemented. Moreover, it is important to highlight that, at present, the studies that used medical imaging techniques for assessing obese individuals were primarily focused on the pelvis and the knee joint; therefore, it is not possible to simply generalize the approach to other body joints or conditions. 

Wearable sensors represent a viable solution to perform movement analysis since they are less expensive and more versatile, with respect to the marker-based optoelectronic stereophotogrammetric systems. Furthermore, they can be applied outside the laboratory in free-living conditions, and can be used in an unrestricted area even for a long acquisition time [15,16,17]. Among wearable sensors, magneto-inertial measurement units (MIMUs) are the most promising ones [83,84]. However, from this systematic review, only five papers employed MIMUs in their study protocol so far. Gait was evaluated using either a single-sensor approach, that was previously validated in healthy and pathological individuals [33,45], or by deploying a 7-sensor setup, specifically addressing lower limbs [49,55,58]. Further, Pamukoff et al. [62] adopted electromagnetic wearable sensors to compare gait biomechanics between normal-weight and obese young adults. In addition to STAs, one of the main sources of uncertainties related to the use of wearable sensors is the presence of kinematics “crosstalk”. This phenomenon can also affect marker-based systems and is linked to the error that can be made in the definition of the rotation axes around which the kinematics are decomposed (e.g., for the knee, the axes of flexion/extension, adduction/abduction, internal/external rotation) [85]; therefore, all the joint angles estimated out-of-sagittal-plane should be carefully interpreted. Moreover, wearable devices are also sensitive to STAs, which were specifically addressed by fixing the sensors over bones instead of muscles and using elastic bands and medical tape [49].

Most of the selected papers investigate the effect of obesity on biomechanics outcomes compared to a control group; unfortunately, only a few studies proposed a coherent approach for the evaluation of motion in obese people including a proper validation. Specifically, on this very topic, Lerner et al. [44] proposed an obese-specific marker-set and compared the output with that obtained from a standard marker-set designed for a normal-weight population. Staying on this subject, Horsak et al. [48] investigated whether test-retest reliability for three-dimensional gait kinematics in a young obese population is affected by the identification of the hip joint center; in particular, the authors assessed either a predictive or a functional hip joint center localization approach. Finally, Agostini et al. [55] validated a gait analysis system based on magneto-inertial sensors, both in normal-weight and overweight/obese subjects; the validation was performed with respect to a reference multichannel recording system providing direct measurements of joint angles in the sagittal plane through electrogoniometers, which indeed present inherent limitations.

From a deeper analysis of the included paper, we identified two main categories of limitations; the former is related to the population specifically involved in the studies, whereas the latter concerns the use of suitable technological solutions.

Concerning the first limitation, most of the evaluated studies involved in fact both males and females introducing a source of potential variability. In fact, obesity modifies the body geometry by adding mass to different regions and dissimilar fat distribution in males and females, and this could produce gender-related effects [33]. Moreover, the current literature did not consider that different body shapes (e.g., apple-shaped vs. pear-shaped) may change the biomechanics output [86]. Another limitation highlighted in the included papers is the absence of a stratification of the participants in terms of the severity of obesity. The lack of homogeneous groups reduced the generalizability of the results, making difficult the comparison among groups since it is not possible to reliably identify which one of these confounding factors directly influenced the measured parameters.

Second, in most of the analyzed articles, the authors focused on the analysis of different biomechanical properties characterizing the obese population, but they did not address the reliability of the technology specifically used to obtain those parameters/metrics. In fact, over 13 years of analyzed literature, only three papers proposed solutions for studying the movement of obese individuals and a proper validation of them; the majority of included papers only narratively referred to some specific limitations that have been noticed during the experimental procedures, but did not report quantitative information and/or possible strategies to mitigate them.

However, we are aware that these limitations could be ascribed to the lack of a non-invasive reliable method for the measurement of the movement specifically designed for obese individuals. Indeed, future studies should consider the use of optimization methods for reducing the influence of STAs, as well as suitable procedures for biomechanical modelling to properly mitigate the errors possibly associated with sensors/markers placement. 

## 5. Conclusions

Obesity inherently requires the need to quantify the functional limitations due to the critical physical conditions. Indeed, the movement analysis through marker-based optoelectronic stereophotogrammetric systems is the most widespread approach in specialized laboratories; however, although it is considered reliable, this solution is particularly affected by soft-tissue artifacts. In recent years, wearable systems have been representing a viable solution to monitor the movement of obese subjects even at home during their daily activities, but the kinematics in the frontal and transversal plane should be carefully interpreted due to the presence of crosstalk. Soft tissue artifacts and kinematic crosstalk are still debated topics in human motion analysis literature, especially for obese subjects. The general problem related to the STAs issue could be addressed by employing medical imaging techniques, which can represent the basis for the validation of more agile solutions. In fact, the proposed medical imaging approaches are expensive, difficult to use due to their inherent complexities, bulky, and resource-consuming.

From this systematic review emerges the urgency for further research to account for the effects of subcutaneous adiposity on kinematic data collection and analysis, and the need for dedicated, feasible, and reliable solutions that may improve the accuracy of the measurements in the obese population.

## Figures and Tables

**Table 2:** List of the included papers assessed using the methodological index (MINORS) tool, to quantify the individual and overall Risk of Bias. Legend of items 1: A clearly stated aim; 2: Inclusion of consecutive patients; 3: Prospective collection of data; 4: Endpoints appropriate to the aim of the study; 5: Unbiased assessment of the study endpoint; 6: Follow-up period appropriate to the aim of the study; 7: Loss to follow-up less than 5%; 8: Prospective calculation of the study size; 9: Adequate control group; 10: Contemporary groups; 11: Baseline equivalence of groups; 12: Adequate statistical analyses. The items are scored 0 (not reported), 1 (reported but inadequate), or 2 (reported and adequate). The global ideal score is 16 for non-comparative studies and 24 for comparative studies.

Author (Year)	1	2	3	4	5	6	7	8	9	10	11	12	Tot. Score
Vakula et al., 2022 [30]	2	1	2	2	0	0	0	2	2	2	2	2	17 (24)
Kim et al., 2022 (b) [29]	2	1	2	2	0	0	0	2	2	2	2	1	16 (24)
Kim et al., 2022 (a) [28]	2	1	2	2	0	0	0	2	2	2	2	1	16 (24)
Pau et al., 2021 [40]	2	1	2	2	0	0	0	2	2	2	2	1	16 (24)
Law et al., 2021 [38]	2	1	2	2	0	0	0	0	2	2	2	1	14 (24)
Kim et al., 2021 [37]	2	1	2	2	0	0	0	2	2	2	2	1	16 (24)
Ghasemi et al., 2021 [7]	2	1	2	2	0	0	0	2	2	2	2	2	17 (24)
Garcia et al., 2021 [34]	2	1	2	2	0	0	0	0	2	2	2	2	15 (24)
Cimolin et al., 2021 [33]	2	1	2	2	0	0	0	2	-	-	-	-	9 (16)
Capodaglio et al., 2021 [31]	2	1	2	2	0	0	0	2	2	1	2	2	16 (24)
Sample et al., 2020 [42]	2	1	2	2	0	0	0	2	2	2	2	2	17 (24)
Maktouf et al., 2020 [41]	2	1	2	2	0	0	0	2	2	2	2	2	17 (24)
Vakula et al., 2019 [35]	2	1	2	2	0	0	0	2	2	2	2	2	17 (24)
Rosso et al., 2019 [49]	2	1	2	2	0	0	0	0	2	1	2	2	14 (24)
Pamukoff et al., 2019 [47]	2	1	2	2	0	0	0	2	2	2	2	2	17 (24)
Dames et al., 2019 [46]	2	1	2	2	0	0	0	2	-	-	-	-	9 (16)
Cimolin et al., 2019 [45]	2	1	2	2	0	0	0	0	2	2	1	2	14 (24)
Badaway et al., 2019 [43]	2	1	2	2	0	0	0	0	2	2	2	2	15 (24)
Yocum et al., 2018 [54]	2	1	2	2	0	0	0	0	2	2	2	1	14 (24)
Milner et al., 2018 [53]	2	1	2	2	0	0	0	2	2	2	2	2	17 (24)
Horsak et al., 2018 [48]	2	1	2	2	0	0	0	0	-	-	-	-	7 (16)
Clément et al., 2018 [20]	2	1	2	2	0	0	0	0	2	2	2	1	14 (24)
Yang et al., 2017 [61]	2	1	2	2	0	0	0	0	2	2	1	1	13 (24)
Sing et al., 2017 [59]	2	1	2	2	0	0	0	0	2	2	2	1	14 (24)
Meng et al., 2017 [58]	2	1	2	2	0	0	0	0	2	2	2	1	14 (24)
Liu et al., 2017 [57]	2	1	2	2	0	0	0	2	2	2	1	1	15 (24)
Camomilla et al., 2017 [21]	2	1	2	2	0	0	0	0	-	-	-	-	7 (16)
Agostini et al., 2017 [55]	2	1	2	2	0	0	0	0	2	2	2	1	14 (24)
Pamukoff et al., 2016 [62]	2	1	2	2	0	0	0	2	2	2	2	2	17 (24)
Sing et al., 2015 [65]	2	1	2	2	0	0	0	1	2	2	1	1	14 (24)
Fu et al., 2015 [64]	2	1	2	2	0	0	0	0	2	1	1	1	12 (24)
Lerner et al., 2014 (b) [44]	2	1	2	2	0	0	0	0	2	1	1	1	12 (24)
Lerner et al., 2014 (a) [69]	2	1	2	2	0	0	0	0	2	1	1	1	12 (24)
Haight et al., 2014 [68]	2	1	2	2	0	0	0	0	2	2	1	1	12 (24)
Glave et al., 2014 [67]	2	1	2	2	0	0	0	2	2	2	2	1	16 (24)
Silvernail et al., 2013 [74]	2	1	2	2	0	0	0	2	2	2	2	1	16 (24)
Russel et al., 2013 [73]	2	1	2	2	0	0	0	2	2	2	2	1	16 (24)
Roemer et al., 2013 [72]	2	1	2	2	0	0	0	2	2	2	2	2	17 (24)
Ranavolo et al., 2013 [71]	2	1	2	2	0	0	0	0	2	2	2	2	15 (24)
Mignardot et al., 2013 [70]	2	1	2	2	0	0	0	0	2	2	2	1	14 (24)
Ehlen et al., 2011 [77]	2	1	2	2	0	0	0	0	-	-	-	-	7 (16)
Vismara et al., 2010 [79]	2	1	2	2	0	0	0	0	2	2	1	2	14 (24)
Russel et al., 2010 [78]	2	1	2	2	0	0	0	2	2	2	2	1	16 (24)

## Data Availability

Not applicable.

## Supplementary Materials

The following supporting information can be downloaded at: https://www.mdpi.com/article/10.3390/s23063175/s1.
