# Supplementary material for: Technological Solutions for Human Movement Analysis in Obese Subjects: A Systematic Review
Source: Sensors (Basel). 2023 Mar 16;23(6):3175. doi: 10.3390/s23063175 (PMC10059733; doi:10.3390/s23063175)
Supplement: Supplementary file 1 [file sensors-23-03175-s001.zip › sensors-2158614-supplementary.pdf]

Serial number	*Reason of exclusion*	*Reference*
1	FULL TEXT NOT AVAILABLE	Alberton, C. L., Fonseca, B. A., Nunes, G. N., Bergamin, M., & Pinto, S. S. (n.d.). Magnitude of vertical ground reaction force during water-based exercises in women with obesity. *SPORTS BIOMECHANICS*. https://doi.org/10.1080/14763141.2021.1872690
2	FULL TEXT NOT AVAILABLE	Almashaqbeh, S., B. Al-Sheikh, W. A. B. Wan Abas, and N. A. Abu Osman. 2015. "Lower Extremities Biomechanics of Regular Stair Climbing: Slim Versus Obese." *Biomedical Engineering - Applications, Basis and Communications* 27 (4). doi:10.4015/S1016237215500362
3	FULL TEXT NOT AVAILABLE	Board, W. J., Reynolds, M. M., Ehlen, K., Reiser, R., & Browning, R. C. (2011). The Effects of Obesity on the Biomechanics of Gradient Walking. *MEDICINE AND SCIENCE IN SPORTS AND EXERCISE*, *43*(5), 29–30. https://doi.org/10.1249/01.MSS.0000402759.22075.75
4	FULL TEXT NOT AVAILABLE	Bobowik, P., & Wiszomirska, I. (2021). The impact of obesity and age on the risk of falls in elderly women. *ACTA OF BIOENGINEERING AND BIOMECHANICS*, *23*(2), 123–130. https://doi.org/10.37190/ABB-01804-2021-03
5	FULL TEXT NOT AVAILABLE	Buckova, K., Lobotková, J., Hirjaková, Z., Bzdúšková, D., & Hlavačka, F. (2014). Postural control assessed by limit of stability in obese adults. *Activitas Nervosa Superior Rediviva*, *56*(3), 87–90. https://www.scopus.com/inward/record.uri?eid=2-s2.0-84922065099&partnerID=40&md5=f14bf78fa0a07795f1eb368d66839b75
6	FULL TEXT NOT AVAILABLE	Cau, N., Cimolin, V., Brugliera, L., Ventura, G., Galli, M., & Capodaglio, P. (2017). Range of motion limitations of the upper body in obese female workers. *MEDICINA DEL LAVORO*, *108*(6), 455–465. https://doi.org/10.23749/mdl.v108i6.6339
7	FULL TEXT NOT AVAILABLE	Choy, K. R., Sin, S., Tong, Y., Udupa, J. K., Luchtenburg, D. M., Wagshul, M. E., Arens, R., & Wootton, D. M. (2021). Upper airway effective compliance during wakefulness and sleep in obese adolescents studied via two-dimensional dynamic MRI and semiautomated image segmentation. *Journal of Applied Physiology (Bethesda, Md. : 1985)*, *131*(2), 532–543. https://doi.org/10.1152/japplphysiol.00839.2020

8	FULL TEXT NOT AVAILABLE	Cimolin, V., Capodaglio, P., Cau, N., Galli, M., Santovito, C., Patrizi, A., Tringali, G., & Sartorio, A. (2017). Computation of spatio-temporal parameters in level walking using a single inertial system in lean and obese adolescents. *Biomedizinische Technik. Biomedical Engineering*, *62*(5), 505–511. https://doi.org/10.1515/bmt-2015-0180
9	FULL TEXT NOT AVAILABLE	Carvalho, F. R. P., da Conceição Figueira Martins, A. T., & Teixeira, A. M. M. B. (2012). Analyses of gait and jump tasks in female obese adolescents. *Pediatric Exercise Science*, *24*(1), 26–33. https://doi.org/10.1123/pes.24.1.26
10	FULL TEXT NOT AVAILABLE	Sousa, A. L., Gabriel, R., Faria, A. M., Aragao, F. R., & Moreira, M. H. R. (2015). Behavior of temporal parameters of the ground reactive forces for the walking of postmenopausal women. *ACTA OF BIOENGINEERING AND BIOMECHANICS*, *17*(3), 119–127. https://doi.org/10.5277/ABB-00122-2014-03
11	FULL TEXT NOT AVAILABLE	Desrochers, P., Ayoub, M., & Gill, S. (2021). Beyond the Biomechanics: How Knee Factors, Physical Activity, Depressed Mood, and Health Modulate the Relationship Between Obesity and Altered Gait. *JOURNAL OF SPORT & EXERCISE PSYCHOLOGY*, *43*, S26–S26.
12	FULL TEXT NOT AVAILABLE	Dunias, P., Gransier, R., Jin, A., Statham, A. & Willems, P. 2011, "Wearable human body joint and posture measuring system", *BIODEVICES 2011 - Proceedings of the International Conference on Biomedical Electronics and Devices*, pp. 268.
13	FULL TEXT NOT AVAILABLE	Gupta, M., Dashottar, A., & Borstad, J. D. (2013). Scapula kinematics differ by body mass index. *Journal of Applied Biomechanics*, *29*(4), 380–385. https://doi.org/10.1123/jab.29.4.380
14	FULL TEXT NOT AVAILABLE	Leidecker, E., Kellermann, P., Tiszberger, M.G., Molics, B., Bohner-Beke, A., Nyárády, J. & Kránicz, J. 2016, "Analysis of plantar pressure patterns among obese population", *Orvosi hetilap*, vol. 157, no. 48, pp. 1919-1925.
15	FULL TEXT NOT AVAILABLE	Merrill, Z., Bova, G., Chambers, A., & Cham, R. (2018). Effect of Trunk Segment Boundary Definitions on Frontal Plane Segment Inertial Calculations. *Journal of Applied Biomechanics*, *34*(3), 232–235. https://doi.org/10.1123/jab.2016-0319
16	FULL TEXT NOT AVAILABLE	Neri, S. G. R., Gadelha, A. B., Correia, A. L. M., Pereira, J. C., de David, A. C., & Lima, R. M. (2017). Obesity is Associated With Altered Plantar Pressure Distribution in Older Women. *JOURNAL OF APPLIED BIOMECHANICS*, *33*(5), 323–329. https://doi.org/10.1123/jab.2016-0357

17	FULL TEXT NOT AVAILABLE	Papapanagiotou, V., Diou, C., Zhou, L., J, van den B., Mars, M., & Delopoulos, A. (2016). A novel approach for chewing detection based on a wearable PPG sensor. *Annual International Conference of the IEEE Engineering in Medicine and Biology Society. IEEE Engineering in Medicine and Biology Society. Annual International Conference*, *2016*, 6485–6488. https://doi.org/10.1109/EMBC.2016.7592214
18	FULL TEXT NOT AVAILABLE	Pau, M., Leban, B., & Pau, M. (2013). Alterations in the plantar pressure patterns of overweight and obese schoolchildren due to backpack carriage. *Journal of the American Podiatric Medical Association*, *103*(4), 306–313. https://doi.org/10.7547/1030306
19	FULL TEXT NOT AVAILABLE	Prachgosin, T., Chong, D. Y., Leelasamran, W., Smithmaitrie, P., & Chatpun, S. (2015). Medial longitudinal arch biomechanics evaluation during gait in subjects with flexible flatfoot. *Acta of Bioengineering and Biomechanics*, *17*(4), 121–130. https://pubmed.ncbi.nlm.nih.gov/26898763/
20	FULL TEXT NOT AVAILABLE	Riddiford-Harland, D. L., Steele, J. R., & Baur, L. A. (2011). Medial midfoot fat pad thickness and plantar pressures: are these related in children? *International Journal of Pediatric Obesity: IJPO : An Official Journal of the International Association for the Study of Obesity*, *6*(3), 261–266. https://doi.org/10.3109/17477166.2011.579974
21	FULL TEXT NOT AVAILABLE	Sathe, N., & Hiwale, A. (2020). *Achieving wellness by monitoring the gait pattern with behavioral intervention for lifestyle diseases* doi:10.1007/978-981-32-9343-4_17
22	FULL TEXT NOT AVAILABLE	Sazonov, E., Browning, R., Sazonova, N., Schutz, Y., & Hill, J. O. (2010). Classification of Physical Activity and Prediction of Energy Expenditure By a Wearable Shoe Sensor. *OBESITY*, *18*, S82–S82.
23	FULL TEXT NOT AVAILABLE	Shultz SP, Sitler MR, Tierney RT, Hillstrom HJ, Song J. Consequences of pediatric obesity on the foot and ankle complex. J Am Podiatr Med Assoc. 2012 Jan-Feb;102(1):5-12. doi: 10.7547/1020005. PMID: 22232315.
24	FULL TEXT NOT AVAILABLE	Silva L., M, B. J., Rattes, C. S., Sayão, L. B., Reinaux, C. A., Campos, S. L., Brandão, D. C., Fregonezi, G., Aliverti, A., & A, D. de A. (2015). The influence of supine posture on chest wall volume changes is higher in obese than in normal weight children. *Applied Physiology, Nutrition, and Metabolism = Physiologie Appliquee, Nutrition et Metabolisme*, *40*(2), 178–183. https://doi.org/10.1139/apnm-2014-0201
25	FULL TEXT NOT AVAILABLE	Singh, B., Francis, S. L., Janz, K. F., & Yack, H. J. (2014). Effect Of Cardiorespiratory Fitness And Fatigue On Gait Biomechanics In Obese Children? *MEDICINE AND*

		SCIENCE IN SPORTS AND EXERCISE, *46*(5), 443–443. https://doi.org/10.1249/01.mss.0000494787.39415.64
26	FULL TEXT NOT AVAILABLE	Singh, B., Niino, M. F., Goulart, J. D., & Hammons, A. (2018). Effect Of Cardiorespiratory Fitness, Fatigue And Muscular Strength On Gait Biomechanics In Obese Children. *MEDICINE AND SCIENCE IN SPORTS AND EXERCISE*, *50*(5), 509–509.
27	FULL TEXT NOT AVAILABLE	Sterner, J. A., Reaves, S. K., Aguinaldo, A. L., Hazelwood, S. J., & Klisch, S. M. (2022). Inverse dynamics analysis of youth pitching arm kinetics using body composition imaging. *SPORTS BIOMECHANICS*, *21*(9), 993–1007. https://doi.org/10.1080/14763141.2020.1715470
28	FULL TEXT NOT AVAILABLE	Ugbolue, U. C., Yates, E. L., Rowland, K. E., Wearing, S. C., Gu, Y., Lam, W. K., Baker, J. S., Sculthorpe, N. F., & Dutheil, F. (2021). A novel simplified biomechanical assessment of the heel pad during foot plantarflexion. *Proceedings of the Institution of Mechanical Engineers. Part H, Journal of Engineering in Medicine*, *235*(2), 197–207. https://doi.org/10.1177/0954411920971069
29	FULL TEXT NOT AVAILABLE	Wang, W., Wang, D. M., & Lai, C. H. (2018). THE THREE-DIMENSIONAL MOVEMENT CORRELATIONS BETWEEN ELBOW AND WRIST JOINT AND ANTHROPOMETRIC DETERMINANTS. *JOURNAL OF MECHANICS IN MEDICINE AND BIOLOGY*, *18*(2). https://doi.org/10.1142/S0219519418500136
30	FULL TEXT NOT AVAILABLE	Yang, H., Fu, C., He, R., & Ma, G. (2022). Impact of overweight and obesity on knee joint biomechanics during running in children. *Chinese Journal of School Health*, *43*(4), 570–577. https://doi.org/10.16835/j.cnki.1000-9817.2022.04.021
31	FULL TEXT NOT AVAILABLE	Zdrodowska, A., Wiszomirska, I., Kaczmarczyk, K., & Kosmol, A. (2018). Effects of anthropometric factors on postural stability in individuals with hearing impairment. *ACTA OF BIOENGINEERING AND BIOMECHANICS*, *20*(1), 109–115. https://doi.org/10.5277/ABB-01014-2017-024
32	FULL TEXT NOT AVAILABLE	Zhang, T., Tang, W., & Sazonov, E. S. (2012). Classification of posture and activities by using decision trees. *Annual International Conference of the IEEE Engineering in Medicine and Biology Society. IEEE Engineering in Medicine and Biology Society. Annual International Conference*, *2012*, 4353–4356. https://doi.org/10.1109/EMBC.2012.6346930

33	POPULATION NOT STRATIFIED OR BMI <30 Kg/m^2	Ahsan M. Determine the kinematics and kinetics parameters associated with bilateral gait patterns among healthy, overweight, and obese adults. Acta Biomed. 2022 Oct 26;93(5):e2022228. doi: 10.23750/abm.v93i5.13060. PMID: 36300229; PMCID: PMC9686171.
34	POPULATION AFFECTED BY PATHOLOGIES OR PAIN	Al Khatib F, Gouissem A, Mbarki R, Adouni M. Biomechanical Characteristics of the Knee Joint during Gait in Obese versus Normal Subjects. Int J Environ Res Public Health. 2022 Jan 16;19(2):989. doi: 10.3390/ijerph19020989. PMID: 35055810; PMCID: PMC8775559.
35	POPULATION <18 YEARS	Allam HH, Muhsen A, Al-Walah MA, Alotaibi AN, Alotaibi SS, Elsayyad LK. Effects of Plyometric Exercises versus Flatfoot Corrective Exercises on Postural Control and Foot Posture in Obese Children with a Flexible Flatfoot. Appl Bionics Biomech. 2021 Oct 31;2021:3635660. doi: 10.1155/2021/3635660. PMID: 34754329; PMCID: PMC8572602.
36	FOCUSED ON POSTURE OR BALANCE OR PLANTAR PRESSURE	Ameer, Mariam A., Alanazi, Munirah S., Alhabbad, Abeer S., Alabas, Ahlam M., Al-Ruwaili, Raghad R., AL-Ruwaili, Saba F., Al-Aljubab, Wasan K., Al-Ruwaili, Taif F., Al-awwad, Ethar I. and Al-Abbad, Ammar M.. "Influence of obesity on spatiotemporal gait parameters among female students from Jouf University, Saudi Arabia" Biomedical Human Kinetics, vol.14, no.1, 2022, pp.127-134. https://doi.org/10.2478/bhk-2022-0016
37	FOCUSED ON POSTURE OR BALANCE OR PLANTAR PRESSURE	Bacha IL, Benetti FA, Greve JM. Baropodometric analyses of patients before and after bariatric surgery. Clinics (Sao Paulo). 2015 Nov;70(11):743-7. doi: 10.6061/clinics/2015(11)05. PMID: 26602521; PMCID: PMC4642488.
38	POPULATION AFFECTED BY PATHOLOGIES OR PAIN	Blazek K, Asay JL, Erhart-Hledik J, Andriacchi T. Adduction moment increases with age in healthy obese individuals. J Orthop Res. 2013 Sep;31(9):1414-22. doi: 10.1002/jor.22390. Epub 2013 Jun 4. PMID: 23737249.
39	POPULATION NOT STRATIFIED OR BMI <30 kg/m^2	Bartels W, Demol J, Gelaude F, Jonkers I, Vander Sloten J. Computed tomography-based joint locations affect calculation of joint moments during gait when compared to scaling approaches. Comput Methods Biomech Biomed Engin. 2015 Aug;18(11):1238-1251. doi: 10.1080/10255842.2014.890186. Epub 2014 Mar 18. PMID: 24641349.
40	INSTRUMENTATION NOT SPECIFIED	Bollinger LM, Walaszek MC, Seay RF, Ransom AL. Knee extensor torque and BMI differently relate to sit-to-stand strategies in obesity. Clin Biomech (Bristol, Avon).

		2019 Feb;62:28-33. doi: 10.1016/j.clinbiomech.2019.01.002. Epub 2019 Jan 12. PMID: 30660055.
41	POPULATION NOT STRATIFIED OR BMI <30 kg/m^2	Bonci T, Camomilla V, Dumas R, Chèze L, Cappozzo A. A soft tissue artefact model driven by proximal and distal joint kinematics. J Biomech. 2014 Jul 18;47(10):2354-61. doi: 10.1016/j.jbiomech.2014.04.029. Epub 2014 Apr 26. PMID: 24818796.
42	SURGICAL INTERVENTION	Bonnefoy-Mazure A, Martz P, Armand S, Sagawa Y Jr, Suva D, Turcot K, Miozzari HH, Lübbeke A. Influence of Body Mass Index on Sagittal Knee Range of Motion and Gait Speed Recovery 1-Year After Total Knee Arthroplasty. J Arthroplasty. 2017 Aug;32(8):2404-2410. doi: 10.1016/j.arth.2017.03.008. Epub 2017 Mar 16. PMID: 28545773.
43	POPULATION NOT STRATIFIED OR BMI <30 kg/m^2	Borhani M, McGregor AH, Bull AM. An alternative technical marker set for the pelvis is more repeatable than the standard pelvic marker set. Gait Posture. 2013 Sep;38(4):1032-7. doi: 10.1016/j.gaitpost.2013.05.019. Epub 2013 Jun 19. PMID: 23790572; PMCID: PMC3989066.
44	POPULATION <18 YEARS	Briggs MS, Spech C, King R, McNally M, Paponetti M, Bout-Tabaku S, Schmitt L. Obese Youth Demonstrate Altered Landing Knee Mechanics Unrelated to Lower-Extremity Peak Torque When Compared With Healthy Weight Youth. J Appl Biomech. 2021 Apr 1;37(2):109-117. doi: 10.1123/jab.2020-0013. Epub 2021 Jan 15. PMID: 33450728.
45	FOCUSED ON POSTURE OR BALANCE OR PLANTAR PRESSURE	Caderby T, Caron N, Verkindt C, Bonazzi B, Dalleau G, Peyrot N. Obesity-related alterations in anticipatory postural mechanisms associated with gait initiation. Exp Brain Res. 2020 Nov;238(11):2557-2567. doi: 10.1007/s00221-020-05914-8. Epub 2020 Sep 2. PMID: 32876708.
46	FOCUSED ON POSTURE OR BALANCE OR PLANTAR PRESSURE	Castro M, Abreu S, Sousa H, Machado L, Santos R, Vilas-Boas JP. Ground reaction forces and plantar pressure distribution during occasional loaded gait. Appl Ergon. 2013 May;44(3):503-9. doi: 10.1016/j.apergo.2012.10.016. Epub 2012 Nov 16. PMID: 23157973
47	FOCUSED ON POSTURE OR BALANCE OR PLANTAR PRESSURE	Cau N, Cimolin V, Galli M, Precilios H, Tacchini E, Santovito C, Capodaglio P. Center of pressure displacements during gait initiation in individuals with obesity. J Neuroeng Rehabil. 2014 May 7;11:82. doi: 10.1186/1743-0003-11-82. PMID: 24885764; PMCID: PMC4026057.

48	PUBLICATION TYPE	G. Cesarelli, L. Donisi, G. Di Caprio, M. Scioli, A. Biancardi and G. D'Addio, "Statistical correlation analysis between kinematic features and clinical indexes and scales for obese patients," *2021 IEEE International Symposium on Medical Measurements and Applications (MeMeA)*, Lausanne, Switzerland, 2021, pp. 1-5, doi: 10.1109/MeMeA52024.2021.9478776.
49	FOCUSED ON POSTURE OR BALANCE OR PLANTAR PRESSURE	Chambers AJ, Sukits AL, McCrory JL, Cham R. The effect of obesity and gender on body segment parameters in older adults. Clin Biomech (Bristol, Avon). 2010 Feb;25(2):131-6. doi: 10.1016/j.clinbiomech.2009.10.015. Epub 2009 Dec 11. PMID: 20005028; PMCID: PMC2820296
50	POPULATION NOT STRATIFIED OR BMI <30 kg/m²	Chehab EF, Andriacchi TP, Favre J. Speed, age, sex, and body mass index provide a rigorous basis for comparing the kinematic and kinetic profiles of the lower extremity during walking. J Biomech. 2017 Jun 14;58:11-20. doi: 10.1016/j.jbiomech.2017.04.014. Epub 2017 Apr 20. PMID: 28501342
51	POPULATION NOT STRATIFIED OR BMI <30 kg/m²	Choi H, Lim J, Lee S. Body fat-related differences in gait parameters and physical fitness level in weight-matched male adults. Clin Biomech (Bristol, Avon). 2021 Jan;81:105243. doi: 10.1016/j.clinbiomech.2020.105243. Epub 2020 Dec 8. PMID: 33309933.
52	SURGICAL INTERVENTION	Christensen JC, Capin JJ, Hinrichs LA, Aljehani M, Stevens-Lapsley JE, Zeni JA. Gait mechanics are influenced by quadriceps strength, age, and sex after total knee arthroplasty. J Orthop Res. 2021 Jul;39(7):1523-1532. doi: 10.1002/jor.24878. Epub 2020 Nov 2. PMID: 33034899; PMCID: PMC8635453.
53	FOCUSED ON POSTURE OR BALANCE OR PLANTAR PRESSURE	Cibulková N, Daďová K, Mašková K, Busch A, Kobesová A, Vařeková J, Hašpicová M, Matoulek M. Bariatric surgery and exercise: A pilot study on postural stability in obese individuals. PLoS One. 2022 Jan 14;17(1):e0262651. doi: 10.1371/journal.pone.0262651. PMID: 35030216; PMCID: PMC8759698.
54	FOCUSED ON POSTURE OR BALANCE OR PLANTAR PRESSURE	Cieślińska-Świder J, Furmanek MP, Błaszczyk JW. The influence of adipose tissue location on postural control. J Biomech. 2017 Jul 26;60:162-169. doi: 10.1016/j.jbiomech.2017.06.027. Epub 2017 Jun 27. PMID: 28705486.
55	POPULATION <18 YEARS	Cilli M, Serbest K, Kayaoglu E. The effect of body weight on joint torques in teenagers: Investigation of sit-to-stand movement. Clinical Biomechanics, Volume 83, 2021, 105288, ISSN 0268-0033, https://doi.org/10.1016/j.clinbiomech.2021.105288.

56	POPULATION AFFECTED BY PATHOLOGIES OR PAIN	Cimolin V, Vismara L, Galli M, Grugni G, Cau N, Capodaglio P. Gait strategy in genetically obese patients: a 7-year follow up. Res Dev Disabil. 2014 Jul;35(7):1501-6. doi: 10.1016/j.ridd.2014.04.005. Epub 2014 Apr 21. PMID: 24763375.
57	FOCUSED ON POSTURE OR BALANCE OR PLANTAR PRESSURE	Clarkson S, Flint SW, Broom DR, Capehorn M, Wheat J. 3D surface-imaging for volumetric measurement in people with obesity. Technol Health Care. 2018;26(2):363-369. doi: 10.3233/THC-171138. PMID: 29332058.
58	POPULATION <18 YEARS	Collado-Mateo, D.; Domínguez-Muñoz, F.J.; Adsuar, J.C.; Parraca, J.A.; Tomas-Carus, P.; Garcia-Gordillo, M.Á.; Raimundo, A.M. Test-Retest Intra-Session Reliability of Isokinetic Knee Strength Measurements in Obese Children. *Appl. Sci.* **2020**, *10*, 5923. https://doi.org/10.3390/app10175923
59	POPULATION <18 YEARS	D'Hondt E, Segers V, Deforche B, Shultz SP, Tanghe A, Gentier I, De Bourdeaudhuij I, De Clercq D, Lenoir M. The role of vision in obese and normal-weight children's gait control. Gait Posture. 2011 Feb;33(2):179-84. doi: 10.1016/j.gaitpost.2010.10.090. Epub 2010 Nov 20. PMID: 21094609.
60	SURGICAL INTERVENTION	De Pieri E, Lunn DE, Chapman GJ, Rasmussen KP, Ferguson SJ, Redmond AC. Patient characteristics affect hip contact forces during gait. Osteoarthritis and Cartilage. Volume 27, Issue 6, 2019, Pages 895-905, ISSN 1063-4584, https://doi.org/10.1016/j.joca.2019.01.016.
61	SURGICAL INTERVENTION	DeVita P, Rider P, Hortobágyi T. Reductions in knee joint forces with weight loss are attenuated by gait adaptations in class III obesity. Gait Posture. 2016 Mar;45:25-30. doi: 10.1016/j.gaitpost.2015.12.040. Epub 2016 Jan 6. PMID: 26979878.
62	FOCUSED ON POSTURE OR BALANCE OR PLANTAR PRESSURE	Desrochers PC, Kim D, Keegan L, Gill SV. Association between the Functional Gait Assessment and spatiotemporal gait parameters in individuals with obesity compared to normal weight controls: A proof-of-concept study. J Musculoskelet Neuronal Interact. 2021 Sep 1;21(3):335-342. PMID: 34465671; PMCID: PMC8426657.
63	FOCUSED ON POSTURE OR BALANCE OR PLANTAR PRESSURE	Diniz-Sousa F, Veras L, Boppre G, Devezas V, Santos-Sousa H, Preto J, Machado L, Vilas-Boas JP, Oliveira J, Fonseca H. How Does Bariatric Surgery Affect Fall Risk Factors? Obes Surg. 2021 Aug;31(8):3506-3513. doi: 10.1007/s11695-021-05400-2. Epub 2021 Apr 2. PMID: 33797733.
64	OTHER FOCUS	Diniz-Sousa F, Veras L, Ribeiro JC, Boppre G, Devezas V, Santos-Sousa H, Preto J, Machado L, Vilas-Boas JP, Oliveira J, Fonseca H. Accelerometry calibration in people with class II-III obesity: Energy expenditure prediction and physical activity

		intensity identification. Gait & Posture, Volume 76, 2020, Pages 104-109, ISSN 0966-6362, https://doi.org/10.1016/j.gaitpost.2019.11.008.
65	OTHER FOCUS	Ellis K, Kerr J, Godbole S, Staudenmayer J, Lanckriet G. Hip and Wrist Accelerometer Algorithms for Free-Living Behavior Classification. Med Sci Sports Exerc. 2016 May;48(5):933-40. doi: 10.1249/MSS.0000000000000840. PMID: 26673126; PMCID: PMC4833514.
66	FOCUSED ON POSTURE OR BALANCE OR PLANTAR PRESSURE	Engelberger RP, Indermühle A, Baumann F, Fahrni J, Diehm N, Kucher N, Egermann U, Laederach K, Baumgartner I, Willenberg T. Diurnal changes of lower leg volume in obese and non-obese subjects. Int J Obes (Lond). 2014 Jun;38(6):801-5. doi: 10.1038/ijo.2013.178. Epub 2013 Sep 13. PMID: 24030515.
67	POPULATION AFFECTED BY PATHOLOGIES OR PAIN	Fang MA, Heiney C, Yentes JM, Harada ND, Masih S, Perell-Gerson KL. Effects of contralateral versus ipsilateral cane use on gait in people with knee osteoarthritis. PM R. 2015 Apr;7(4):400-6. doi: 10.1016/j.pmrj.2014.09.018. Epub 2014 Oct 8. PMID: 25305371.
68	OTHER FOCUS	Feito Y, Bassett DR, Tyo B, Thompson DL. Effects of body mass index and tilt angle on output of two wearable activity monitors. Med Sci Sports Exerc. 2011 May;43(5):861-6. doi: 10.1249/MSS.0b013e3181fefd40. PMID: 20962689.
69	POPULATION NOT STRATIFIED OR BMI <30 kg/m^2	Fischer AG, Wolf A. The effects of body weight unloading on kinetics and muscle activity of overweight males during Overground walking. Clinical Biomechanics, Volume 52, 2018, Pages 80-85, ISSN 0268-0033, https://doi.org/10.1016/j.clinbiomech.2018.01.013.
70	FOCUSED ON POSTURE OR BALANCE OR PLANTAR PRESSURE	Fourchet F, Maffiuletti NA, Agosti F, Patrizi A, Sartorio A. Impact of rocker sole footwear on plantar pressure distribution during standing and walking in adult obese women. Disabil Rehabil. 2020 Apr;42(7):927-930. doi: 10.1080/09638288.2018.1512012. Epub 2018 Nov 25. PMID: 30474431.
71	FOCUSED ON POSTURE OR BALANCE OR PLANTAR PRESSURE	Frames CW, Soangra R, Lockhart TE, Lach J, Ha DS, Roberto KA, Lieberman A. Dynamical Properties of Postural Control in Obese Community-Dwelling Older Adults †. Sensors (Basel). 2018 May 24;18(6):1692. doi: 10.3390/s18061692. PMID: 29794998; PMCID: PMC6021983.
72	POPULATION NOT STRATIFIED OR BMI <30 kg/m^2	Galli M, Cimolin V, Ferrario D, Patti P, Heaney G, Freedland R, Albertini G, Brown WT. Quantitative 3D evaluation of step ascent and descent in individuals with Down syndrome--analysis of a daily challenging task. J Intellect Disabil Res. 2013

		Dec;57(12):1143-51. doi: 10.1111/j.1365-2788.2012.01627.x. Epub 2012 Sep 24. PMID: 22998578.
73	POPULATION NOT STRATIFIED OR BMI <30 kg/m^2	Gao X, Wang L, Shen F, Ma Y, Fan Y, Niu H. Dynamic walking stability of elderly people with various BMIs. Gait Posture. 2019 Feb;68:168-173. doi: 10.1016/j.gaitpost.2018.11.027. Epub 2018 Nov 20. PMID: 30497036.
74	POPULATION NOT STRATIFIED OR BMI <30 kg/m^2	Glave AP, Olson JM, Applegate DK, Brezzo RD. The effects of two different arm positions and weight status on select kinematic variables during the bodyweight squat. J Strength Cond Res. 2012 Nov;26(11):3148-54. doi: 10.1519/JSC.0b013e318243fefb. PMID: 22158095.
75	POPULATION NOT STRATIFIED OR BMI <30 kg/m^2	Golightly YM, Hannan MT, Dufour AB, Hillstrom HJ, Jordan JM. Foot disorders associated with overpronated and oversupinated foot function: the Johnston County osteoarthritis project. Foot Ankle Int. 2014 Nov;35(11):1159-65. doi: 10.1177/1071100714543907. Epub 2014 Jul 18. PMID: 25037712; PMCID: PMC4392721.
76	OTHER FOCUS	Gonzalez M, Gates DH, Rosenblatt NJ. The impact of obesity on gait stability in older adults. J Biomech. 2020 Feb 13;100:109585. doi: 10.1016/j.jbiomech.2019.109585. Epub 2019 Dec 17. PMID: 31911052; PMCID: PMC7061260.
77	POPULATION NOT STRATIFIED OR BMI <30 kg/m^2	Haggerty M, Dickin DC, Popp J, Wang H. The influence of incline walking on joint mechanics. Gait Posture. 2014 Apr;39(4):1017-21. doi: 10.1016/j.gaitpost.2013.12.027. Epub 2014 Jan 8. PMID: 24472218
78	POPULATION AFFECTED BY PATHOLOGIES OR PAIN	Hall M, Bennell KL, Beavers DP, Wrigley TV, DeVita P, Messier SP. Does frontal knee kinematics predict treatment outcomes? Exploratory analyses from the Intensive Diet and Exercise for Arthritis (IDEA) trial. Gait Posture. 2018 Jun;63:139-144. doi: 10.1016/j.gaitpost.2018.04.045. Epub 2018 Apr 27. PMID: 29730489
79	OTHER FOCUS	Hamilton M, Strawderman L, Hale B, Babski-Reeves K. Effects of BMI and task parameters on postural sway during simulated small parts assembly. Ergonomics. 2015;58(3):504-12. doi: 10.1080/00140139.2014.972468. Epub 2014 Oct 24. PMID: 25343497.
80	PUBLICATION TYPE	Handrigan GA, Corbeil P, Simoneau M, Teasdale N. Balance control is altered in obese individuals. J Biomech. 2010 Jan 19;43(2):383-4; author reply 385-6. doi: 10.1016/j.jbiomech.2009.08.041. Epub 2009 Oct 31. PMID: 19880125.

81	POPULATION AFFECTED BY PATHOLOGIES OR PAIN	Harding GT, Hubley-Kozey CL, Dunbar MJ, Stanish WD, Astephen Wilson JL. Body mass index affects knee joint mechanics during gait differently with and without moderate knee osteoarthritis. Osteoarthritis Cartilage. 2012 Nov;20(11):1234-42. doi: 10.1016/j.joca.2012.08.004. Epub 2012 Aug 16. PMID: 22902710.
82	POPULATION AFFECTED BY PATHOLOGIES OR PAIN	Harding GT, Dunbar MJ, Hubley-Kozey CL, Stanish WD, Astephen Wilson JL. Obesity is associated with higher absolute tibiofemoral contact and muscle forces during gait with and without knee osteoarthritis. Clin Biomech (Bristol, Avon). 2016 Jan;31:79-86. doi: 10.1016/j.clinbiomech.2015.09.017. Epub 2015 Sep 30. Erratum in: Clin Biomech (Bristol, Avon). 2017 Mar;43:121. PMID: 26476602.
83	POPULATION NOT STRATIFIED OR BMI <30 kg/m^2	Herssens N, van Criekinge T, Saeys W, Truijen S, Vereeck L, van Rompaey V, Hallemans A. 2020 An investigation of the spatio-temporal parameters of gait and margins of stability throughout adulthood. J. R. Soc. Interface 17: 20200194. http://dx.doi.org/10.1098/rsif.2020.0194
84	POPULATION NOT STRATIFIED OR BMI <30 kg/m^2	Hora M, Sládek V, Soumar L, Stráníková K, Michálek T ."Influence of body mass and lower limb length on knee flexion angle during walking in humans," Folia Zoologica, 61(3–4), 330-339, (1 November 2012)
85	POPULATION <18 YEARS	Horsak B, Pobatschnig B, Baca A, Greber-Platzer S, Kreissl A, Nehrer S, Wondrasch B, Crevenna R, Keilani M, Kranzl A. Within-assessor reliability and minimal detectable change of gait kinematics in a young obese demographic. Gait Posture. 2017 May;54:112-118. doi: 10.1016/j.gaitpost.2017.02.028. Epub 2017 Mar 2. PMID: 28288331.
86	POPULATION <18 YEARS	Horsak B, Schwab C, Clemens C, Baca A, Greber-Platzer S, Kreissl A, Kranzl A. Is the reliability of 3D kinematics of young obese participants dependent on the hip joint center localization method used?, Gait & Posture, Volume 59, 2018, Pages 65-70, ISSN 0966-6362, https://doi.org/10.1016/j.gaitpost.2017.09.029.
87	POPULATION <18 YEARS	Horsak B, Schwab C, Baca A, Greber-Platzer S, Kreissl A, Nehrer S, Keilani M, Crevenna R, Kranzl A, Wondrasch B. Effects of a lower extremity exercise program on gait biomechanics and clinical outcomes in children and adolescents with obesity: A randomized controlled trial. Gait Posture. 2019 May;70:122-129. doi: 10.1016/j.gaitpost.2019.02.032. Epub 2019 Feb 27. PMID: 30851623.

88	POPULATION >18 YEARS	Horsak B, Schwab C, Durstberger S, Thajer A, Greber-Platzer S, Kainz H, Jonkers I, Kranzl A. 3D free-hand ultrasound to register anatomical landmarks at the pelvis and localize the hip joint center in lean and obese individuals. Sci Rep. 2021 May 20;11(1):10650. doi: 10.1038/s41598-021-89763-7. PMID: 34017023; PMCID: PMC8170673.
89	POPULATION <18 YEARS	Horsak B, Schwab C, Leboeuf F, Kranzl A. Reliability of walking and stair climbing kinematics in a young obese population using a standard kinematic and the CGM2 model. Gait Posture. 2021 Jan;83:96-99. doi: 10.1016/j.gaitpost.2020.10.017. Epub 2020 Oct 16. PMID: 33129173.
90	SURGICAL INTERVENTION	Hortobágyi T, Herring C, Pories WJ, Rider P, Devita P. Massive weight loss-induced mechanical plasticity in obese gait. J Appl Physiol (1985). 2011 Nov;111(5):1391-9. doi: 10.1152/japplphysiol.00291.2011. Epub 2011 Aug 18. PMID: 21852410; PMCID: PMC3220312.
91	POPULATION NOT STRATIFIED OR BMI <30 kg/m^2	Huffman KD, Sanford BA, Zucker-Levin AR, Williams JL, Mihalko WM. Increased hip abduction in high body mass index subjects during sit-to-stand. Gait Posture. 2015 Feb;41(2):640-5. doi: 10.1016/j.gaitpost.2015.01.014. Epub 2015 Jan 21. PMID: 25655834.
92	POPULATION <18 YEARS	Hung YC, Mangiafreno M, Gill SV. Whole body organization during a symmetric bimanual pick up task in overweight and obese children. Gait Posture. 2017 Feb;52:95-99. doi: 10.1016/j.gaitpost.2016.11.030. Epub 2016 Nov 19. PMID: 27888697.
93	OTHER FOCUS	Jalai CM, Diebo BG, Cruz DL, Poorman GW, Vira S, Buckland AJ, Lafage R, Bess S, Errico TJ, Lafage V, Passias PG. The impact of obesity on compensatory mechanisms in response to progressive sagittal malalignment. Spine J. 2017 May;17(5):681-688. doi: 10.1016/j.spinee.2016.11.016. Epub 2016 Dec 1. PMID: 27916684.
94	POPULATION NOT STRATIFIED OR BMI <30 kg/m^2	Jeong H, Johnson AW, Feland JB, Petersen SR, Staten JM, Bruening DA (2021) Added body mass alters plantar shear stresses, postural control, and gait kinetics: Implications for obesity. PLoS ONE 16(2): e0246605. https://doi.org/10.1371/journal.pone.0246605
95	POPULATION NOT STRATIFIED OR BMI <30 kg/m^2	Kathirgamanathan B, Silva P, Fernandez J (2019) Implication of obesity on motion, posture and internal stress of the foot: an experimental and finite element analysis, Computer Methods in Biomechanics and Biomedical Engineering, 22:1, 47-54, DOI: 10.1080/10255842.2018.1527320

96	POPULATION AFFECTED BY PATHOLOGIES OR PAIN	Kean CO, Bennell KL, Wrigley TV, Hinman RS. Modified walking shoes for knee osteoarthritis: Mechanisms for reductions in the knee adduction moment. J Biomech. 2013 Aug 9;46(12):2060-6. doi: 10.1016/j.jbiomech.2013.05.011. Epub 2013 Jun 14. PMID: 23768609.
97	POPULATION NOT STRATIFIED OR BMI <30 kg/m^2	Kim HK, Dai X, Lu SH, Lu TW, Chou LS. Discriminating features of ground reaction forces in overweight old and young adults during walking using functional principal component analysis, Gait & Posture, Volume 94, 2022, Pages 166-172, ISSN 0966-6362, https://doi.org/10.1016/j.gaitpost.2022.03.012.
98	POPULATION AFFECTED BY PATHOLOGIES OR PAIN	Knarr BA, Higginson JS, Zeni JA. Change in knee contact force with simulated change in body weight. Comput Methods Biomech Biomed Engin. 2016 Feb;19(3):320-323. doi: 10.1080/10255842.2015.1018193. Epub 2015 Mar 11. PMID: 25760517; PMCID: PMC4567548.
99	POPULATION AFFECTED BY PATHOLOGIES OR PAIN	Ko S, Stenholm S, Ferrucci L. Characteristic gait patterns in older adults with obesity--results from the Baltimore Longitudinal Study of Aging. J Biomech. 2010 Apr 19;43(6):1104-10. doi: 10.1016/j.jbiomech.2009.12.004. Epub 2010 Jan 18. PMID: 20080238; PMCID: PMC2849896.
100	FOCUSED ON POSTURE OR BALANCE OR PLANTAR PRESSURE	Ku PX, Abu Osman NA, Yusof A, Wan Abas WA. Biomechanical evaluation of the relationship between postural control and body mass index. J Biomech. 2012 Jun 1;45(9):1638-42. doi: 10.1016/j.jbiomech.2012.03.029. Epub 2012 Apr 14. PMID: 22507349.
101	INSTRUMENTATION NOT SPECIFIED	Lathrop-Lambach RL, Asay JL, Jamison ST, Pan X, Schmitt LC, Blazek K, Siston RA, Andriacchi TP, Chaudhari AM. Evidence for joint moment asymmetry in healthy populations during gait. Gait Posture. 2014 Sep;40(4):526-31. doi: 10.1016/j.gaitpost.2014.06.010. Epub 2014 Jul 1. PMID: 25035185; PMCID: PMC4267535.
102	POPULATION NOT STRATIFIED OR BMI <30 kg/m^2	Lee Y, Shin S. The Effect of Body Composition on Gait Variability Varies with Age: Interaction by Hierarchical Moderated Regression Analysis. Int J Environ Res Public Health. 2022 Jan 21;19(3):1171. doi: 10.3390/ijerph19031171. PMID: 35162200; PMCID: PMC8834456.
103	SURGICAL INTERVENTION	Legrand T, Richard V, Bonnefoy-Mazure A, Armand S, Miozzari HH, Turcot K. The impact of body-mass index on the frontal knee alignment estimation using three-

		dimensional reconstruction based on movement analysis. Knee. 2020 Jan;27(1):89-94. doi: 10.1016/j.knee.2019.09.020. Epub 2019 Dec 24. PMID: 31870700.
104	POPULATION NOT STRATIFIED OR BMI <30 kg/m^2	Leirós-Rodríguez, R.; Romo-Pérez, V.; Arce-Fariña, M.E. y García-Soidán, J.L. (2018) Relación entre composición corporal y movimientos realizados durante la marcha en mujeres / Associations between body composition and movements during gait in women. Revista Internacional de Medicina y Ciencias de la Actividad Física y el Deporte vol. 18 (72) pp. 693-707 Http://cdeporte.rediris.es/revista/revista72/artrelacion970.htm
105	POPULATION <18 YEARS	Lerner ZF, Board WJ, Browning RC. Pediatric obesity and walking duration increase medial tibiofemoral compartment contact forces. J Orthop Res. 2016 Jan;34(1):97-105. doi: 10.1002/jor.23028. Epub 2015 Aug 28. PMID: 26271943.
106	POPULATION <18 YEARS	Lerner ZF, Browning RC. Compressive and shear hip joint contact forces are affected by pediatric obesity during walking. J Biomech. 2016 Jun 14;49(9):1547-1553. doi: 10.1016/j.jbiomech.2016.03.033. Epub 2016 Mar 25. PMID: 27040390; PMCID: PMC4885751.
107	POPULATION NOT STRATIFIED OR BMI <30 kg/m^2	Lerner ZF, Haight DJ, DeMers MS, Board WJ, Browning RC. The effects of walking speed on tibiofemoral loading estimated via musculoskeletal modeling. J Appl Biomech. 2014 Apr;30(2):197-205. doi: 10.1123/jab.2012-0206. Epub 2013 Jul 22. PMID: 23878264; PMCID: PMC4423394.
108	POPULATION AFFECTED BY PATHOLOGIES OR PAIN	Li JS, Tsai TY, Felson DT, Li G, Lewis CL. Six degree-of-freedom knee joint kinematics in obese individuals with knee pain during gait. PLoS One. 2017 Mar 24;12(3):e0174663. doi: 10.1371/journal.pone.0174663. Erratum in: PLoS One. 2019 Feb 22;14(2):e0213084. PMID: 28339477; PMCID: PMC5365132.
109	POPULATION AFFECTED BY PATHOLOGIES OR PAIN	Li JS, Tsai TY, Clancy MM, Li G, Lewis CL, Felson DT. Weight loss changed gait kinematics in individuals with obesity and knee pain. Gait Posture. 2019 Feb;68:461-465. doi: 10.1016/j.gaitpost.2018.12.031. Epub 2018 Dec 24. PMID: 30611976; PMCID: PMC6599530.
110	POPULATION AFFECTED BY PATHOLOGIES OR PAIN	Li JS, Tsai TY, Clancy MM, Lewis CL, Felson DT, Li G. Cartilage contact characteristics of the knee during gait in individuals with obesity. J Orthop Res. 2022 Nov;40(11):2480-2487. doi: 10.1002/jor.25288. Epub 2022 Feb 4. PMID: 35076128; PMCID: PMC9309196.

111	POPULATION <18 YEARS	Long JT, Neogi S, Caldwell CM, DeLange MP. Variation inflation factor-based regression modeling of anthropometric measures and temporal-spatial performance: Modeling approach and implications for clinical utility. Clinical Biomechanics, Volume 51, 2018, Pages 51-57, ISSN 0268-0033, https://doi.org/10.1016/j.clinbiomech.2017.11.008.
112	SURGICAL INTERVENTION	Lugade V, Wu A, Jewett B, Collis D, Chou LS. Gait asymmetry following an anterior and anterolateral approach to total hip arthroplasty. Clin Biomech (Bristol, Avon). 2010 Aug;25(7):675-80. doi: 10.1016/j.clinbiomech.2010.05.003. Epub 2010 Jun 9. PMID: 20542608.
113	FOCUSED ON POSTURE OR BALANCE OR PLANTAR PRESSURE	Maffiuletti NA, Visscher R, De Col A, Sartorio A. Differences in soleus H-reflex to M-wave ratio between obese and non-obese individuals. Clin Biomech (Bristol, Avon). 2021 Apr;84:105322. doi: 10.1016/j.clinbiomech.2021.105322. Epub 2021 Mar 11. PMID: 33756402.
114	POPULATION <18 YEARS	Mahaffey R, Morrison SC, Bassett P, Drechsler WI, Cramp MC. Biomechanical characteristics of lower limb gait waveforms: Associations with body fat in children. Gait Posture. 2018 Mar;61:220-225. doi: 10.1016/j.gaitpost.2018.01.019. Epub 2018 Mar 20. PMID: 29413788.
115	POPULATION <18 YEARS	Mahaffey R, Morrison SC, Bassett P, Drechsler WI, Cramp MC. The impact of body fat on three dimensional motion of the paediatric foot during walking. Gait Posture. 2016 Feb;44:155-60. doi: 10.1016/j.gaitpost.2015.12.009. Epub 2015 Dec 14. PMID: 27004650.
116	FOCUSED ON POSTURE OR BALANCE OR PLANTAR PRESSURE	Maktouf W, Guilherme C, Boyas S, Beaune B, Durand S. Relationships between lower limbs fatigability threshold and postural control in obese adults. J Biomech. 2020 May 22;105:109819. doi: 10.1016/j.jbiomech.2020.109819. Epub 2020 Apr 25. PMID: 32423537.
117	POPULATION AFFECTED BY PATHOLOGIES OR PAIN	Malatesta D, Vismara L, Menegoni F, Grugni G, Capodaglio P. Effect of obesity onset on pendular energy transduction at spontaneous walking speed: Prader-Willi versus nonsyndromal obese individuals. Obesity (Silver Spring). 2013 Dec;21(12):E586-91. doi: 10.1002/oby.20455. Epub 2013 Jun 13. PMID: 23554340.
118	SURGICAL INTERVENTION	Martz P, Bourredjem A, Maillefert JF, Binquet C, Baulot E, Ornetti P, Laroche D. Influence of body mass index on sagittal hip range of motion and gait speed

		recovery six months after total hip arthroplasty. Int Orthop. 2019 Nov;43(11):2447-2455. doi: 10.1007/s00264-018-4250-y. Epub 2019 Jan 5. PMID: 30612173.
119	FOCUSED ON POSTURE OR BALANCE OR PLANTAR PRESSURE	Matrangola SL, Madigan ML. The effects of obesity on balance recovery using an ankle strategy. Hum Mov Sci. 2011 Jun;30(3):584-95. doi: 10.1016/j.humov.2010.11.007. Epub 2011 Apr 5. PMID: 21470705.
120	POPULATION NOT STRATIFIED OR BMI <30 kg/m^2	Matuszewska A, Syczewska M. Analysis of the movements of the upper extremities during gait: Their role for the dynamic balance. Gait Posture. 2022 Dec 6;100:82-90. doi: 10.1016/j.gaitpost.2022.12.004. Epub ahead of print. PMID: 36502665.
121	POPULATION <18 YEARS	McMillan AG, Pulver AM, Collier DN, Williams DS. Sagittal and frontal plane joint mechanics throughout the stance phase of walking in adolescents who are obese. Gait Posture. 2010 Jun;32(2):263-8. doi: 10.1016/j.gaitpost.2010.05.008. Epub 2010 Jun 22. PMID: 20573511.
122	POPULATION <18 YEARS	McMillan AG, Phillips KA, Collier DN, Blaise Williams DS. Frontal and sagittal plane biomechanics during drop jump landing in boys who are obese. Pediatr Phys Ther. 2010 Spring;22(1):34-41. doi: 10.1097/PEP.0b013e3181cd1868. PMID: 20142703
123	OTHER FOCUS	Meng H, Gorniak SL. Effects of adiposity on postural control and cognition in older adults. Gait Posture. 2020 Oct;82:147-152. doi: 10.1016/j.gaitpost.2020.09.004. Epub 2020 Sep 6. PMID: 32927221.
124	POPULATION NOT STRATIFIED OR BMI <30 kg/m^2	Merlo A, Campanini I. Impact of instrumental analysis of stiff knee gait on treatment appropriateness and associated costs in stroke patients. Gait Posture. 2019 Jul;72:195-201. doi: 10.1016/j.gaitpost.2019.06.009. Epub 2019 Jun 13. PMID: 31228856.
125	POPULATION NOT STRATIFIED OR BMI <30 kg/m^2	Messier SP, Legault C, Loeser RF, Van Arsdale SJ, Davis C, Ettinger WH, DeVita P. Does high weight loss in older adults with knee osteoarthritis affect bone-on-bone joint loads and muscle forces during walking? Osteoarthritis Cartilage. 2011 Mar;19(3):272-80. doi: 10.1016/j.joca.2010.11.010. Epub 2010 Dec 4. PMID: 21134477; PMCID: PMC3444807.
126	POPULATION AFFECTED BY PATHOLOGIES OR PAIN	Messier SP, Mihalko SL, Legault C, Miller GD, Nicklas BJ, DeVita P, Beavers DP, Hunter DJ, Lyles MF, Eckstein F, Williamson JD, Carr JJ, Guermazi A, Loeser RF. Effects of intensive diet and exercise on knee joint loads, inflammation, and clinical outcomes among overweight and obese adults with knee osteoarthritis: the IDEA randomized clinical trial. JAMA. 2013 Sep 25;310(12):1263-73. doi: 10.1001/jama.2013.277669. PMID: 24065013; PMCID: PMC4450354.

127	POPULATION AFFECTED BY PATHOLOGIES OR PAIN	Messier SP, Pater M, Beavers DP, Legault C, Loeser RF, Hunter DJ, DeVita P. Influences of alignment and obesity on knee joint loading in osteoarthritic gait. Osteoarthritis Cartilage. 2014 Jul;22(7):912-7. doi: 10.1016/j.joca.2014.05.013. Epub 2014 May 21. PMID: 24857973; PMCID: PMC4108726.
128	FOCUSED ON POSTURE OR BALANCE OR PLANTAR PRESSURE	Miller EM , Matrangola SL, Madigan ML. (2011) Effects of obesity on balance recovery from small postural perturbations, Ergonomics, 54:6, 547-554, DOI: 10.1080/00140139.2011.582959
129	POPULATION <18 YEARS	Molina-Garcia P, Plaza-Florido A, Mora-Gonzalez J, Torres-Lopez LV, Vanrenterghem J, Ortega FB. Role of physical fitness and functional movement in the body posture of children with overweight/obesity. Gait Posture. 2020 Jul;80:331-338. doi: 10.1016/j.gaitpost.2020.04.001. Epub 2020 Apr 11. PMID: 32599548.
130	POPULATION <18 YEARS	Molina-Garcia P, Molina-Molina A, Smeets A, Migueles JH, Ortega FB, Vanrenterghem J. Effects of integrative neuromuscular training on the gait biomechanics of children with overweight and obesity. Scand J Med Sci Sports. 2022 Jul;32(7):1119-1130. doi: 10.1111/sms.14163. Epub 2022 Apr 29. PMID: 35398912; PMCID: PMC9540886
131	POPULATION <18 YEARS	Molina-Garcia P, Miranda-Aparicio D, Molina-Molina A, Plaza-Florido A, Migueles JH, Mora-Gonzalez J, Cadenas-Sanchez C, Esteban-Cornejo I, Rodriguez-Ayllon M, Solis-Urra P, Vanrenterghem J, Ortega FB. Effects of Exercise on Plantar Pressure during Walking in Children with Overweight/Obesity. Med Sci Sports Exerc. 2020 Mar;52(3):654-662. doi: 10.1249/MSS.0000000000002157. PMID: 3152482
132	POPULATION <18 YEARS	Monte A. Insight into the biomechanics and bioenergetics of human walking: Obese versus healthy children. Exp Physiol. 2020 Jul;105(7):1058-1059. doi: 10.1113/EP088772. Epub 2020 Jun 3. PMID: 32441828.
133	OTHER FOCUS	Monteiro M, Gabriel R, Aranha J, Neves e Castro M, Sousa M, Moreira M. Influence of obesity and sarcopenic obesity on plantar pressure of postmenopausal women. Clin Biomech (Bristol, Avon). 2010 Jun;25(5):461-7. doi: 10.1016/j.clinbiomech.2010.01.017. Epub 2010 Feb 21. PMID: 20176421.
134	POPULATION <18 YEARS	Montes-Alguacil J, Páez-Moguer J, Jiménez Cebrián AM, Muñoz BÁ, Gijón-Noguerón G, Morales-Asencio JM. The influence of childhood obesity on spatio-temporal gait parameters. Gait Posture. 2019 Jun;71:69-73. doi: 10.1016/j.gaitpost.2019.03.031. Epub 2019 Mar 30. PMID: 31009919.

135	POPULATION NOT STRATIFIED OR BMI <30 kg/m^2	Mullerpatan RP, Agarwal BM, Shetty T, Nehete GR, Narasipura OS. Kinematics of Suryanamaskar Using Three-Dimensional Motion Capture. Int J Yoga. 2019 May-Aug;12(2):124-131. doi: 10.4103/ijoy.IJOY_26_18. PMID: 31143020; PMCID: PMC6521759.
136	FOCUSED ON POSTURE OR BALANCE OR PLANTAR PRESSURE	Naderi A, Baloochi R, Rostami KD, Fourchet F, Degens H. Obesity and foot muscle strength are associated with high dynamic plantar pressure during running. Foot (Edinb). 2020 Sep;44:101683. doi: 10.1016/j.foot.2020.101683. Epub 2020 Apr 3. PMID: 32801069.
137	PUBLICATION TYPE	Hyejeong Nam, Jin-Hyun Kim and Jee-In Kim, "Smart Belt : A wearable device for managing abdominal obesity," *2016 International Conference on Big Data and Smart Computing (BigComp)*, Hong Kong, 2016, pp. 430-434, doi: 10.1109/BIGCOMP.2016.7425964.
138	POPULATION NOT STRATIFIED OR BMI <30 kg/m^2	O'Brien DL, Tyndyk M. Effect of arch type and Body Mass Index on plantar pressure distribution during stance phase of gait. Acta Bioeng Biomech. 2014;16(2):131-5. PMID: 25088082.
139	POPULATION >18 YEARS	Orantes-Gonzalez E, Heredia-Jimenez J. Does schoolbag carriage equally affect obese/overweight and healthy-weight children? Appl Ergon. 2021 Jan;90:103236. doi: 10.1016/j.apergo.2020.103236. Epub 2020 Aug 24. PMID: 32854066.
140	POPULATION NOT STRATIFIED OR BMI <30 kg/m^2	Pamukoff DN, Holmes SC, Garcia SA, Vakula MN, Shumski EJ, Moffit TJ. Influence of body mass index and anterior cruciate ligament reconstruction on gait biomechanics. J Orthop Res. 2022 Oct 7. doi: 10.1002/jor.25451. Epub ahead of print. PMID: 36205181.
141	FOCUSED ON POSTURE OR BALANCE OR PLANTAR PRESSURE	Pamukoff DN, Dudley RI, Vakula MN, Blackburn JT. An evaluation of the heel strike transient in obese young adults during walking gait. Gait Posture. 2016 Sep;49:181-183. doi: 10.1016/j.gaitpost.2016.07.001. Epub 2016 Jul 1. PMID: 27434487.
142	POPULATION <18 YEAR	Pande A, Mohapatra P, Nicorici A, Han JJ. Machine Learning to Improve Energy Expenditure Estimation in Children With Disabilities: A Pilot Study in Duchenne Muscular Dystrophy. JMIR Rehabil Assist Technol. 2016 Jul 19;3(2):e7. doi: 10.2196/rehab.4340. PMID: 28582264; PMCID: PMC5454548.
143	POPULATION AFFECTED BY PATHOLOGIES OR PAIN	Paterson KL, Sosdian L, Hinman RS, Wrigley TV, Kasza J, Dowsey M, Choong P, Bennell KL. Effects of sex and obesity on gait biomechanics before and six months after total knee arthroplasty: A longitudinal cohort study. Gait Posture. 2018

		Mar;61:263-268. doi: 10.1016/j.gaitpost.2018.01.014. Epub 2018 Mar 20. PMID: 29413795.
144	POPULATION AFFECTED BY PATHOLOGIES OR PAIN	Paterson KL, Sosdian L, Hinman RS, Wrigley TV, Kasza J, Dowsey M, Choong P, Bennell KL. The influence of sex and obesity on gait biomechanics in people with severe knee osteoarthritis scheduled for arthroplasty. Clin Biomech (Bristol, Avon). 2017 Nov;49:72-77. doi: 10.1016/j.clinbiomech.2017.08.013. Epub 2017 Sep 5. PMID: 28892670.
145	FOCUSED ON POSTURE OR BALANCE OR PLANTAR PRESSURE	Peduzzi de Castro M, Abreu S, Pinto V, Santos R, Machado L, Vaz M, Vilas-Boas JP. Influence of pressure-relief insoles developed for loaded gait (backpackers and obese people) on plantar pressure distribution and ground reaction forces. Appl Ergon. 2014 Jul;45(4):1028-34. doi: 10.1016/j.apergo.2014.01.005. Epub 2014 Jan 24. PMID: 24468683.
146	POPULATION <18 YEARS	Peyrot N, Morin JB, Thivel D, Isacco L, Taillardat M, Belli A, Duche P. Mechanical work and metabolic cost of walking after weight loss in obese adolescents. Med Sci Sports Exerc. 2010 Oct;42(10):1914-22. doi: 10.1249/MSS.0b013e3181da8d1e. PMID: 20216466.
147	OTHER FOCUS	Pryce R, Kriellaars D. Body segment inertial parameters and low back load in individuals with central adiposity. J Biomech. 2014 Sep 22;47(12):3080-6. doi: 10.1016/j.jbiomech.2014.06.038. Epub 2014 Jul 5. PMID: 25047741.
148	POPULATION NOT STRATIFIED OR BMI <30 kg/m^2	Ransom AL, Walaszek MC, Shapiro R, Bollinger LM. External loading alters lower extremity kinetics, kinematics, and muscle activity in a distribution-specific manner during the transition from stair descent to level walking. Clin Biomech (Bristol, Avon). 2019 Oct;69:71-78. doi: 10.1016/j.clinbiomech.2019.07.008. Epub 2019 Jul 5. PMID: 31302492.
149	POPULATION AFFECTED BY PATHOLOGIES OR PAIN	Robbins SM, Birmingham TB, Maly MR, Chesworth BM, Giffin JR. Comparative diagnostic accuracy of knee adduction moments in knee osteoarthritis: a case for not normalizing to body size. J Biomech. 2011 Mar 15;44(5):968-71. doi: 10.1016/j.jbiomech.2010.12.021. PMID: 21296354.
150	POPULATION NOT STRATIFIED OR BMI <30 kg/m^2	Garman CR, Franck CT, Nussbaum MA, Madigan ML. A bootstrapping method to assess the influence of age, obesity, gender, and gait speed on probability of tripping as a function of obstacle height. J Biomech. 2015 Apr 13;48(6):1229-32. doi: 10.1016/j.jbiomech.2015.01.031. Epub 2015 Feb 3. PMID: 25683521.

151	POPULATION AFFECTED BY PATHOLOGIES OR PAIN	Russell EM, Hamill J. Lateral wedges decrease biomechanical risk factors for knee osteoarthritis in obese women. J Biomech. 2011 Aug 11;44(12):2286-91. doi: 10.1016/j.jbiomech.2011.05.033. Epub 2011 Jun 29. PMID: 21719018.
152	POPULATION NOT STRATIFIED OR BMI <30 kg/m^2	Sazonov ES, Fulk G, Hill J, Schutz Y and Browning R. "Monitoring of Posture Allocations and Activities by a Shoe-Based Wearable Sensor," in *IEEE Transactions on Biomedical Engineering*, vol. 58, no. 4, pp. 983-990, April 2011, doi: 10.1109/TBME.2010.2046738
153	OTHER FOCUS	Schmid S, Armand S, Pataky Z, Golay A, Allet L. The relationship between different body mass index categories and chair rise performance in adult women. J Appl Biomech. 2013 Dec;29(6):705-11. doi: 10.1123/jab.29.6.705. Epub 2013 Feb 19. PMID: 23434794.
154	NO ENGLISH	SCHUTZ, G.R.; DETANICO, D.; GOULART JR., R. y DOS SANTOS, S.G.. Análisis de la biomecánica de la marcha en mujeres con hipertrofia mamaria. *Cir. plást. iberolatinoam.* [online]. 2013, vol.39, n.4 [citado 2023-02-21], pp.369-380. ISSN 1989-2055. https://dx.doi.org/10.4321/S0376-78922013000400007.
155	POPULATION NOT STRATIFIED OR BMI <30 kg/m^2	Sheehan KJ, Gormley J. The influence of excess body mass on adult gait. Clin Biomech (Bristol, Avon). 2013 Mar;28(3):337-43. doi: 10.1016/j.clinbiomech.2013.01.007. Epub 2013 Feb 4. PMID: 23380662.
156	POPULATION <18 YEARS	Shultz SP, Hills AP, Sitler MR, Hillstrom HJ. Body size and walking cadence affect lower extremity joint power in children's gait. Gait Posture. 2010 Jun;32(2):248-52. doi: 10.1016/j.gaitpost.2010.05.001. Epub 2010 May 31. PMID: 20570152.
157	POPULATION <18 YEARS	Shultz SP, D'Hondt E, Lenoir M, Fink PW, Hills AP. The role of excess mass in the adaptation of children's gait. Hum Mov Sci. 2014 Aug;36:12-9. doi: 10.1016/j.humov.2014.05.002. Epub 2014 Jun 6. PMID: 24908185.
158	POPULATION <18 YEARS	Shultz SP, D'Hondt E, Fink PW, Lenoir M, Hills AP. The effects of pediatric obesity on dynamic joint malalignment during gait. Clin Biomech (Bristol, Avon). 2014 Aug;29(7):835-8. doi: 10.1016/j.clinbiomech.2014.05.004. Epub 2014 May 21. PMID: 24889987.
159	POPULATION NOT STRATIFIED OR BMI <30 kg/m^2	Sikandar T, Rabbi MF, Ghazali KH, Altwijri O, Almijalli M, Ahamed NU. Evaluating the difference in walk patterns among normal-weight and overweight/obese individuals in real-world surfaces using statistical analysis and deep learning methods with inertial measurement unit data. Phys Eng Sci Med. 2022

		Dec;45(4):1289-1300. doi: 10.1007/s13246-022-01195-3. Epub 2022 Nov 9. PMID: 36352317
160	FOCUSED ON POSTURE OR BALANCE OR PLANTAR PRESSURE	Silva D, Gabriel R, Moreira M, Abrantes J, Faria A (2016) Foot Rollover Temporal Parameters During Straight-Ahead and Side-Cut Walking in Obese and Nonobese Postmenopausal Women, Journal of Motor Behavior, 48:5, 413-423, DOI:10.1080/00222895.2015.1123139
161	POPULATION <18 YEARS	Singh B, Negatu MG, Francis SL, Janz KF, Yack HJ. Do fitness and fatigue affect gait biomechanics in overweight and obese children? Gait Posture. 2016 Oct;50:190-195. doi: 10.1016/j.gaitpost.2016.09.006. Epub 2016 Sep 8. PMID: 27637091.
162	OTHER FOCUS	Singh B, Brown TD, Callaghan JJ, Yack HJ. Abdomen-thigh contact during forward reaching tasks in obese individuals. J Appl Biomech. 2013 Oct;29(5):517-24. doi: 10.1123/jab.29.5.517. Epub 2012 Nov 21. PMID: 23183157.
163	POPULATION <18 YEARS	Song Q, Yu B, Zhang C, Sun W, Mao D. (2014)<br>Effects of Backpack Weight on Posture, Gait Patterns and Ground Reaction Forces of Male Children with Obesity during Stair Descent, Research in Sports Medicine, 22:2, 172-184, DOI: 10.1080/15438627.2014.881823
164	FOCUSED ON POSTURE OR BALANCE OR PLANTAR PRESSURE	Song J, Kane R, Tango DN, Veur SS, Furmato J, Komaroff E, Foster GD. Effects of weight loss on foot structure and function in obese adults: a pilot randomized controlled trial. Gait Posture. 2015 Jan;41(1):86-92. doi: 10.1016/j.gaitpost.2014.08.013. Epub 2014 Sep 6. PMID: 25245307; PMCID: PMC4297201.
165	POPULATION <18 YEARS	Yan SH, Zhang K, Tan GQ, Yang J, Liu ZC. Effects of obesity on dynamic plantar pressure distribution in Chinese prepubescent children during walking. Gait Posture. 2013 Jan;37(1):37-42. doi: 10.1016/j.gaitpost.2012.05.018. Epub 2012 Jul 31. PMID: 22858245.
166	POPULATION <18 YEARS	Song-Hua Y, Lu W, Kuan Z. Effects of different movement modes on plantar pressure distribution patterns in obese and non-obese Chinese children. Gait Posture. 2017 Sep;57:28-34. doi: 10.1016/j.gaitpost.2017.05.001. Epub 2017 May 4. PMID: 28551468
167	POPULATION <18 YEARS	da Rocha ES, Bratz DT, Gubert LC, de David A, Carpes FP. Obese children experience higher plantar pressure and lower foot sensitivity than non-obese. Clin Biomech (Bristol, Avon). 2014 Aug;29(7):822-7. doi: 10.1016/j.clinbiomech.2014.05.006. Epub 2014 May 28. PMID: 24913089

168	POPULATION <18 YEARS	Steinberg N, Rubinstein M, Nemet D, Ayalon M, Zeev A, Pantanowitz M, Brosh T, Eliakim A. Effects of a Program for Improving Biomechanical Characteristics During Walking and Running in Children Who Are Obese. Pediatr Phys Ther. 2017 Oct;29(4):330-340. doi: 10.1097/PEP.0000000000000440. PMID: 28953178.
169	POPULATION AFFECTED BY PATHOLOGIES OR PAIN	Stewart S, Morpeth T, Dalbeth N, Vandal AC, Carroll M, Davidtz L, Mawston G, Otter S, Rome K. Foot-related pain and disability and spatiotemporal parameters of gait during self-selected and fast walking speeds in people with gout: A two-arm cross sectional study. Gait Posture. 2016 Feb;44:18-22. doi: 10.1016/j.gaitpost.2015.11.004. Epub 2015 Nov 12. PMID: 27004627.
170	POPULATION <18 YEARS	Strutzenberger G, Richter A, Schneider M, Mündermann A, Schwameder H. Effects of obesity on the biomechanics of stair-walking in children. Gait Posture. 2011 May;34(1):119-25. doi: 10.1016/j.gaitpost.2011.03.025. Epub 2011 May 4. PMID: 21536442.
171	POPULATION <18 YEARS	Summa S, De Peppo F, Petrarca M, Caccamo R, Carbonetti R, Castelli E, Ottavio Adorisio D. Gait changes after weight loss on adolescent with severe obesity after sleeve gastrectomy. Surg Obes Relat Dis. 2019 Mar;15(3):374-381. doi: 10.1016/j.soard.2019.01.007. Epub 2019 Jan 24. PMID: 30738737.
172	POPULATION AFFECTED BY PATHOLOGIES OR PAIN	Tassani S, Tio L, Castro-Domínguez F, Monfort J, Monllau JC, González Ballester MA, Noailly J. Relationship Between the Choice of Clinical Treatment, Gait Functionality and Kinetics in Patients With Comparable Knee Osteoarthritis. Front Bioeng Biotechnol. 2022 Mar 11;10:820186. doi: 10.3389/fbioe.2022.820186. PMID: 35360402; PMCID: PMC8962661.
173	PUBLICATION TYPE	David V. Thiel, Hugo G. Espinosa, Glen M. Davis, Elizabeth Dylke, Nasim Foroughi, Sharon L. Kilbreath. Arm Movement: The Effect of Obesity on Active Lifestyles, Procedia Engineering, Volume 60, 2013, Pages 182-187, ISSN 1877-7058, https://doi.org/10.1016/j.proeng.2013.07.033.
174	POPULATION NOT STRATIFIED OR BMI <30 kg/m^2	Urbanek JK, Roth DL, Karas M, Wanigatunga AA, Mitchell CM, Juraschek SP, Cai Y, Appel LJ, Schrack JA. Free-living gait cadence measured by wearable accelerometer: a promising alternative to traditional measures of mobility for assessing fall risk. J Gerontol A Biol Sci Med Sci. 2022 Jan 13:glac013. doi: 10.1093/gerona/glac013. Epub ahead of print. PMID: 35029661.

175	POPULATION AFFECTED BY PATHOLOGIES OR PAIN	Vangeneugden J, Verlaan L, Oomen P, Liu WY, Peters M, Natour N, Emans P, Meijer K. Signatures of knee osteoarthritis in women in the temporal and fractal dynamics of human gait. Clin Biomech (Bristol, Avon). 2020 Jun;76:105016. doi: 10.1016/j.clinbiomech.2020.105016. Epub 2020 Apr 28. PMID: 32438265.
176	OTHER FOCUS	Vargas CB, Picolli F, Dani C, Padoin AV, Mottin CC. Functioning of obese individuals in pre- and postoperative periods of bariatric surgery. Obes Surg. 2013 Oct;23(10):1590-5. doi: 10.1007/s11695-013-0924-0. PMID: 23515976.
177	SURGICAL INTERVENTION	Vartiainen P, Bragge T, Lyytinen T, Hakkarainen M, Karjalainen PA, Arokoski JP. Kinematic and kinetic changes in obese gait in bariatric surgery-induced weight loss, Journal of Biomechanics, Volume 45, Issue 10, 2012, Pages 1769-1774, ISSN 0021-9290, https://doi.org/10.1016/j.jbiomech.2012.05.002.
178	POPULATION NOT STRATIFIED OR BMI <30 kg/m^2	Veras, L., Diniz-Sousa, F., Boppre, G. *et al.* Accelerometer-based prediction of skeletal mechanical loading during walking in normal weight to severely obese subjects. *Osteoporos Int* **31**, 1239–1250 (2020). https://doi.org/10.1007/s00198-020-05295-2
179	OTHER FOCUS	Veras L, Diniz-Sousa F, Boppre G, Moutinho-Ribeiro E, Resende-Coelho A, Devezas V, Santos-Sousa H, Preto J, Vilas-Boas JP, Machado L, Oliveira J, Fonseca H. Mechanical loading prediction through accelerometry data during walking and running. Eur J Sport Sci. 2022 Jul 15:1-18. doi: 10.1080/17461391.2022.2102437. Epub ahead of print. PMID: 35838070.
180	POPULATION <18 YEARS	Villarrasa-Sapiña I, Serra-Añó P, Pardo-Ibáñez A, Gonzalez LM, García-Massó X. Relationship between body composition and vertical ground reaction forces in obese children when walking. Clin Biomech (Bristol, Avon). 2017 Jan;41:77-81. doi: 10.1016/j.clinbiomech.2016.12.008. Epub 2016 Dec 15. PMID: 28012303.
181	STUDY DESIGN	Vismara L, Cimolin V, Menegoni F, Zaina F, Galli M, Negrini S, Villa V, Capodaglio P. Osteopathic manipulative treatment in obese patients with chronic low back pain: a pilot study. Man Ther. 2012 Oct;17(5):451-5. doi: 10.1016/j.math.2012.05.002. Epub 2012 May 31. PMID: 22658268.
182	POPULATION NOT STRATIFIED OR BMI <30 kg/m^2	Walaszek MC, Ransom AL, Capehart S, Pohl MB, Shapiro R, Bollinger LM. External loading alters trunk kinematics and lower extremity muscle activity in a distribution-specific manner during sitting and rising from a chair. J Electromyogr

		Kinesiol. 2017 Jun;34:102-108. doi: 10.1016/j.jelekin.2017.04.005. Epub 2017 Apr 23. PMID: 28460239.
183	POPULATION NOT STRATIFIED OR BMI <30 kg/m^2	Westlake CG, Milner CE, Zhang S, Fitzhugh EC. Do thigh circumference and mass changes alter knee biomechanics during walking? Gait Posture. 2013 Mar;37(3):359-62. doi: 10.1016/j.gaitpost.2012.07.031. Epub 2012 Aug 29. PMID: 22939753.
184	OTHER FOCUS	Whittaker RL, Vidt ME, Lockley RME, Mourtzakis M, Dickerson CR. Upper extremity and trunk body segment parameters are affected by BMI and sex. J Biomech. 2021 Mar 5;117:110230. doi: 10.1016/j.jbiomech.2021.110230. Epub 2021 Jan 9. PMID: 33493714.
185	POPULATION <18 YEARS	Wiesinger HP, Buchecker M, Müller E, Stöggl T, Birklbauer J. Decreased Postural Complexity in Overweight to Obese Children and Adolescents: A Cross-Sectional Study. Front Hum Neurosci. 2022 Apr 28;16:850548. doi: 10.3389/fnhum.2022.850548. PMID: 35572009; PMCID: PMC9097216.
186	PUBLICATION TYPE	Wu X, Yeoh HT, Soangra R, Lockhart TE. Investigation into the Functional Mobility Difference between Obese and Non-Obese Elderly. Proceedings of the Human Factors and Ergonomics Society Annual Meeting. 1 settembre 2012;56(1):1814–6.
187	OTHER FOCUS	Wu X, Lockhart TE, Yeoh HT. Effects of obesity on slip-induced fall risks among young male adults. J Biomech. 2012 Apr 5;45(6):1042-7. doi: 10.1016/j.jbiomech.2011.12.021. Epub 2012 Feb 2. PMID: 22304846; PMCID: PMC3310324.
188	PUBLICATION TYPE	Z. Zhang and G. -Z. Yang, "Monitoring cardio-respiratory and posture movements during sleep: What can be achieved by a single motion sensor," *2015 IEEE 12th International Conference on Wearable and Implantable Body Sensor Networks (BSN)*, Cambridge, MA, USA, 2015, pp. 1-6, doi: 10.1109/BSN.2015.7299409.
189	POPULATION NOT STRATIFIED OR BMI <30 kg/m^2	**Glave, A. P.**, Di Brezzo, R., Applegate, D. K., & Olson, J.M. (2013). The effects of weight and activity on select kinematic gait variables in adult females. *Journal of Physical Education and Sport, 13*(4), 471-478. doi: **10.7752/jpes.2013.04075**
